# Effect of Vacuum Annealing Temperature on Optoelectrical Performance of IGZTO Films and Thin-Film Transistors †

**DOI:** 10.3390/ma19214671

**Published:** 2026-11-02

**Authors:** Weicheng Cao, Liang Fang, Xia Zhao, Guoqing Zhang, Gaobin Liu, Fang Wu, Dan Liu, Wensi Cai, Qiang Li

**Affiliations:** 1 Chongqing Key Laboratory of Interface Physics in Energy Conversion, College of Physics, Chongqing University, Chongqing 400044, China; 2 Key Laboratory of Optoelectronic Technology & Systems (Ministry of Education), College of Optoelectronic Engineering, Chongqing University, Chongqing 400044, China; 3 Chongqing Youth Vocational & Technical College, Chongqing 400712, China; 4 Chongqing BOE Display Technology Co., Ltd., Chongqing 400700, China; 5 Zhongshan Zhilong New Materials Technology Co., Ltd., Zhongshan 528459, China

**Keywords:** indium gallium zinc tin oxide (IGZTO), vacuum annealing, thin-film transistor, magnetron sputtering, annealing temperature, amorphous oxide semiconductors (AOS)

## Abstract

Thin-film transistors (TFTs) based on quinary indium-gallium-zinc-tin oxide (IGZTO) were fabricated via radio frequency (RF) magnetron sputtering. The impact of vacuum annealing temperatures (25–450 °C) on the microstructural and optoelectrical properties of films and TFTs was systematically investigated. All IGZTO thin films are amorphous and transparent, with optical bandgaps ranging from 3.22 eV to 3.63 eV. The one annealed at 350 °C exhibited the smoothest surface. The carrier concentrations and Hall mobilities of IGZTO films vary with the annealing temperature in the range of 7.44 × 10^18^ ~ 6.37 × 10^19^ cm^−3^ and 20.89~35.53 cm^2^·V^−1^·s^−1^, respectively. Oxygen vacancies ($V_{O}^{\times }$) are considered the major origin of the conductivity of IGZTO films, and their evolution with annealing temperatures was elucidated. The TFT annealed at 350 °C achieved a saturation mobility (*μ_sat_*) of 19.7 cm^2^ V^−1^ s^−1^, threshold voltage (*V_TH_*) of 4.72 V, subthreshold swing (*SS*) of 0.56 V dec^−1^, and *I_on_/I_off_* of 8.1 × 10^7^, but TFTs annealed at other temperatures show no switching behaviour. TCAD simulations indicate that vacuum annealing elevates $V_{O}^{\times }$ density, especially at the film surface; the switching failure at 150 °C and 450 °C is attributed to the pronounced structural disorder expressed by large characteristic slope energies of tail states, while the failure at 250 °C is due to excessive $V_{O}^{\times }$ density. This work may provide useful guidance for the performance optimisation of IGZTO film and TFTs via annealing.

## 1. Introduction

Amorphous oxide semiconductors (AOSs) have been widely investigated as channel materials of thin-film transistors (TFTs) for large-area electronics, such as flat-panel displays, because they provide good uniformity over large substrates, allow low processing temperatures and offer a tunable carrier concentration [1,2,3]. Amorphous In–Ga–Zn–O (a-IGZO) has received particular attention since Nomura et al. demonstrated room-temperature-fabricated transparent flexible TFTs based on this material in 2004 [4], and it is now regarded as one of the most promising alternatives to hydrogenated amorphous silicon (a-Si:H) [2,3]. As display panels move towards larger substrates, finer pixel pitches and higher frame rates, however, the field-effect mobility and the reliability of a-IGZO devices may fall short of the requirements of these applications [3,5]. Among the approaches explored to overcome this limitation, the addition of Sn to the a-IGZO matrix leads to the quinary amorphous In–Ga–Zn–Sn–O (a-IGZTO) system [6,7]. In a-IGZTO, the Sn 5s orbitals are reported to overlap with the In 5s orbitals and to contribute to a more dispersive conduction band, which is expected to favour electron transport [6]. Moreover, the Sn–O bond is considered to be stronger than the In–O bond, which has been suggested to suppress the formation of weak oxygen bonds [6,7]. Accordingly, several groups have reported higher mobilities and improved stability for a-IGZTO than for a-IGZO TFTs, and field-effect mobilities of the TFT above 90 cm^2^ V^−1^ s^−1^ have been reported [7], while the reliability of IGZTO devices under bias stress has also been examined [8]. Cation substitution has additionally been used to modify the carrier concentration and the stability of a-IGZO-based channels, for example with Mo, Nd or Sn [6,9,10].

Post-deposition thermal treatment is widely used to densify sputtered oxide films and to reduce the structural disorder introduced during growth [11]. For AOSs, the usable annealing window is relatively narrow, since at low temperature, the amorphous network is only partially relaxed, and point defects remain, whereas at higher temperature, crystallization or void formation may degrade the film [11,12]. The annealing ambient is also known to affect the defect chemistry, and both oxidizing (O_2_) and inert (N_2_) atmospheres have been employed to tune the electrical properties of oxide TFTs, including atomic-layer-deposited zinc–tin–oxide devices [13,14,15]. Vacuum annealing has attracted interest in this context because the low oxygen partial pressure favours the release of weakly bonded oxygen from the film, which offers a route to control the concentration and the depth distribution of oxygen vacancies [16,17]. A large part of these annealing studies concerns quaternary systems, such as a-IGZO, a-In–Ga–Sn–O and a-In–Zn–Sn–O [13,14,18,19,20,21], whereas only a smaller number of reports deal with the quinary a-IGZTO system [7,8,22,23,24]. Choi et al. examined how the annealing atmosphere influences the performance and the bias stability of Ga-doped InZnSnO TFTs [18]. Li et al. followed the evolution of IGZTO films annealed in air [22]. Park et al. tuned the threshold voltage of IGZTO TFTs through deep-ultraviolet exposure and the post-treatment temperature [23] and reported that oxygen annealing affects both the electrical performance and the stability of Sn-doped oxide TFTs [25]. Reports on vacuum-annealed a-IGZTO films and on the TFTs made from them are, by comparison, still scarce, and the transfer characteristics that have been published differ appreciably, so that the origin of these differences remains under discussion [14,16,17,18,26].

In our previous studies, quinary IGZTO thin films were grown by RF magnetron sputtering; the deposition parameters were optimized [27], and the influence of the annealing temperature in air on the microstructure and the optoelectronic properties of the films was examined [22]. Vacuum annealing was not part of either of these studies, and the electrical characteristics of the corresponding TFTs were not addressed there. The present work continues this line of study and replaces air with vacuum as the annealing ambient, in order to examine how the vacuum annealing temperature affects the optical and electrical properties of a-IGZTO films and of bottom-gate TFTs fabricated from the same layers. In addition, a computational approach was employed in order to explore the underlying microscopic mechanisms, which can provide valuable insight into the relationships among defect chemistry, the electronic density of states (DOS) and device performance in multicomponent semiconductors [8]. Systematically comparing and analysing the experimental and numerically simulated electrical properties of IGZTO film or TFTs is still an innovative task [28,29].

The present study follows the evolution of a-IGZTO layers annealed in vacuum between room temperature (RT) and 450 °C. Optical transmittance, Hall transport, surface topography and the local chemical environment of the films were characterized in sequence, and the origin of the observed trends is discussed. TFTs were then fabricated from the same layers, their figures of merit were extracted from the measured transfer curves (part of these device data was reported in a conference digest [30]), and the annealing temperature giving the best combination of mobility, subthreshold swing and switching ratio was identified together with the possible defect-related mechanism. Finally, TCAD simulations were used to test the proposed defect picture and to follow how the density of gap states evolves with the annealing temperature. The results may help to define a practical process window for a-IGZTO channels and to rationalize some of the inconsistencies among the reported data.

## 2. Experimental Details

### 2.1. Film Preparation

The films, nominally 50 nm thick, were deposited by radio-frequency (RF) magnetron sputtering of a ceramic target supplied by Zhongshan Zhilong New Material Technology Co., Ltd. (Zhongshan, China). The atomic ratio was In:Ga:Zn:Sn = 1:0.5:1.5:0.25, and the oxygen content was left unconstrained (x). Sputtering took place in pure Ar at a flow rate of 40 sccm and an RF power of 90 W; the chamber was pumped to a base pressure of 5 × 10^−4^ Pa and the working pressure during growth was kept at 0.8 Pa. The as-grown films were subsequently annealed for 1 h in a tube furnace evacuated to 4 × 10^−2^ Pa at 25, 150, 250, 350 or 450 °C.

### 2.2. Device Fabrication

**Figure 1:**
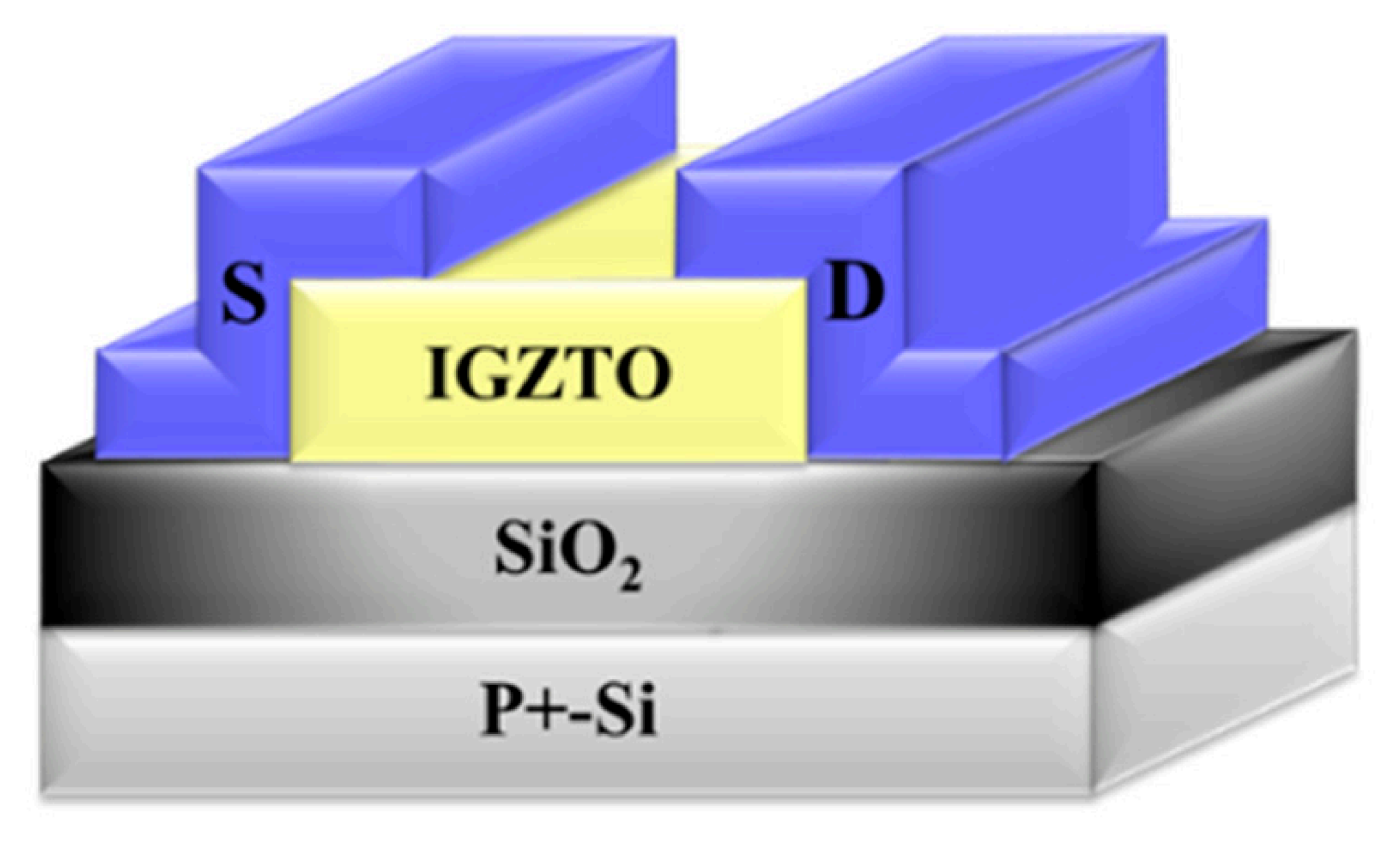
Schematic device structure of a-IGZTO TFTs.

Bottom-gate, staggered transistors were built on heavily doped p-type Si wafers that also served as the gate electrode (Figure 1). A 200 nm SiO_2_ layer grown thermally on the wafer acted as the gate insulator, the 50 nm IGZTO channel was deposited under the conditions given in Section 2.1, and the source/drain (S/D) pads consisted of 100 nm Al deposited by direct-current (DC) magnetron sputtering. Both the channel island and the S/D electrodes were defined by metal shadow masks, which produced devices with a channel width W of 1000 μm and a channel length L of 300 μm. The completed transistors were annealed for 1 h in the same evacuated tube furnace (4 × 10^−2^ Pa) at 25, 150, 250, 350 or 450 °C; the 25 °C devices were simply kept at room temperature after fabrication and serve as the as-prepared reference.

### 2.3. Characterization and Measurement

Phase constitution was checked by X-ray diffraction (XRD, Bruker D8 Advance, Bruker Corporation, Billerica, MA, USA). Surface topography was imaged by scanning electron microscopy (SEM, Tescan Mira 3 LMH, Tescan Group, Brno, Czech Republic) and quantified by atomic force microscopy (AFM, Oxford Instruments MFP-3D-BIO, Oxford Instruments, Oxford, UK). Optical transmittance and absorbance were recorded on a UV-Vis spectrophotometer (Shimadzu UV-3600, Shimadzu Corporation, Kyoto, Japan), the free-carrier concentration, Hall mobility and resistivity were obtained with a Hall measurement system (Ecopia HMS-3000, Ecopia Co., Ltd., Anyang, Republic of Korea), and the chemical states were probed by X-ray photoelectron spectroscopy (XPS, Thermo Fisher ESCALAB 250Xi, Thermo Fisher Scientific, Waltham, MA, USA). The transfer and output curves of the devices were recorded on a Keithley 4200-SCS parameter analzer (Tektronix, Beaverton, OR, USA).

In detail, XRD measurements were performed in grazing-incidence geometry using Ni-filtered Cu Kα radiation (λ = 1.5418 Å) at a fixed incidence angle of 1°, over a 2θ range of 15–80°. Surface morphologies were examined by SEM using secondary-electron imaging at an accelerating voltage of 7 kV and a nominal magnification of 33,500×. AFM observation was conducted over a scan area of 5 μm × 5 μm for each sample. Optical transmittance spectra were recorded by UV–Vis spectroscopy over the wavelength range of 300–800 nm, with a wavelength sampling interval of 1 nm. XPS measurements were performed using a monochromatic Al Kα excitation source (1486.6 eV). The analysis was conducted with a photoelectron take-off angle of 90° relative to the sample surface normal, and the analyzed area was an elliptical spot of approximately 500 μm × 500 μm. The base pressure of the analysis chamber was maintained below 5 × 10^−9^ mbar during all measurements. Prior to sample testing, the binding energy scale of the spectrometer was calibrated using standard reference samples: the Au 4f_7_/_2_ peak at 83.96 eV, Ag 3d_5_/_2_ peak at 368.21 eV and Cu 2p_3_/_2_ peak at 932.62 eV. A low-energy electron flood gun (charge neutralizer) was employed to compensate for surface charging on the insulating samples. No Ar^+^ sputter etching was performed on the sample surfaces before analysis to avoid altering the original surface chemical states. Calibration of binding energies was performed with the C 1s peak set to 284.8 eV [26].

TFT transfer characteristics were measured at RT in a light-shielded enclosure. The drain–source voltage (*V_DS_*) was fixed at 30 V, while the gate–source voltage (*V_GS_*) was swept from −20 to 30 V and then back to −20 V in steps of 0.5 V. Bias-stress stability measurements were performed at room temperature in a light-shielded enclosure. Constant gate–source voltages (*V_GS_*) of +20 and −20 V were applied for positive bias stress (PBS) and negative bias stress (NBS), respectively, for a total stress duration of 3600 s. Transfer characteristics were recorded before stress application and at stress-time intervals of 600 s. The threshold-voltage shift was defined as Δ*V_TH_* = *V_TH_*(*t*) − *V_TH_*(0), where *V_TH_*(0) and *V_TH_*(*t*) denote the threshold voltages before stressing and after a stress duration of t, respectively.

### 2.4. Performance Parameter Extraction

The transmittance and absorbance data of IGZTO films were obtained from UV-Vis measurements, and the optical band gap (*E_g_*) could be calculated on the basis of the film transmittance. Previous studies have reported that IGZTO is a direct band gap semiconductor [31]. Combining with its transmittance spectra, the optical absorption coefficient *α* can be calculated by Equation (1), and then *E_g_* is derived using Equation (2).

(1) $$\begin{array}{c} \alpha =-\frac{1}{d}\ln\left(T\right) \end{array}$$

(2) $$\begin{array}{c} {\left(\mathrm{\alpha hv}\right)}^{2}=C\left(\mathrm{hv}-E_{g}\right) \end{array}$$

where α stands for the absorption coefficient, d for the film thickness and T for the transmittance, *hv* is the photon energy and C an empirical constant [32]. In the specific calculation, the obtained (*αhv*)^2^ values were plotted against *hv*, and linear fitting was performed in the region with the maximum slope of the curve. The value corresponding to the intersection of the fitted straight line with the horizontal axis (*hv* axis) is defined as the *E_g_* of the IGZTO films.

The Urbach energy (*E_u_*) is usually used to represent the degree of disorder of a material, which directly mirrors the characteristic slope of band-tail states: a higher *E_u_* value corresponds to a wider band-tail distribution. Since band-tail states arise from structural disorder, the *E_u_* shows a negative correlation with structural order—a smaller *E_u_* indicates a better structural order and lower defect density, and vice versa [33]. *E_u_* can be calculated from the optical absorption data of IGZTO thin films. Specifically, *E_u_* is derived by rearranging Equation (3) into Equation (4):

(3) $$\begin{array}{c} \alpha =\alpha_{0}\times \exp\left(\frac{\mathrm{h\upsilon}-E_{g}}{E_{U}}\right) \end{array}$$

(4) $$\begin{array}{c} \mathrm{ln\alpha}=\ln\alpha_{0}+\frac{1}{E_{U}}\left(\mathrm{h\upsilon}-E_{g}\right) \end{array}$$

where *α* is the absorption coefficient, *α*_0_ is a constant, *hv* is the photon energy, and *E_g_* is the optical bandgap [33]. Eu then follows from the inverse slope of the linear section of the *lnα*-*hυ* plot.

The current–voltage (I–V) behaviour of each device was recorded with a semiconductor parameter analyzer, from which the threshold voltage (*V*_TH_), the on/off current ratio (*I*_on_/*I*_off_), the saturation mobility (*μ*_Sat_) and the subthreshold swing (*SS*) were extracted. *V_TH_* was obtained by linearly extrapolating the saturation-regime branch of the $\sqrt{I_{\mathrm{DS}}}-V_{\mathrm{GS}}$ curve to its intersection with the *V*_GS_ axis, where *I*_DS_ and *V*_GS_ denote the drain–source and gate–source voltages, respectively. The ratio *I*_on_/*I*_off_ compares the drain current at *V_GS_* = 20 V with that at *V_GS_* = −5 V. *μ*_Sat_ and *SS* follow from Equations (5) and (6) [34]:

(5) $$\begin{array}{c} \mu_{\mathrm{Sat}}=\frac{2Lk^{2}}{WC_{\mathrm{OX}}} \end{array}$$

(6) $$\begin{array}{c} \mathrm{SS}={\left(\frac{\mathrm{dlog}\left(I_{\mathrm{DS}}\right)}{dV_{\mathrm{GS}}}\right)}^{-1} \end{array}$$

in which k = $d\sqrt{I_{\mathrm{DS}}}/dV_{\mathrm{GS}}$ is the slope term obtained from the $\sqrt{I_{\mathrm{DS}}}$–*V*_GS_ characteristic, *L* and *W* are the channel length and width, and *C_OX_* stands for the areal capacitance of the 200 nm SiO_2_ gate dielectric.

### 2.5. TCAD Simulation Setup

To thoroughly investigate the effects of vacuum annealing on the microscopic carrier transport mechanism and defect behaviour in IGZTO TFTs, TCAD simulations were performed to characterize the electrical properties of the devices by solving Poisson’s equation and the charge neutrality condition. The structure of the simulated IGZTO TFT is identical to the experimentally fabricated device, which is depicted in Figure 1.

The defect states were modeled based on Equations (7)–(11) as follows [28,29]:

(7) $$\begin{array}{c} g_{\mathrm{TA}}(E)=N_{\mathrm{TA}}\exp(\frac{E-E_{C}}{W_{\mathrm{TA}}}) \end{array}$$

(8) $$\begin{array}{c} g_{\mathrm{TD}}(E)=N_{\mathrm{TD}}\exp(\frac{E_{V}-E}{W_{\mathrm{TD}}}) \end{array}$$

(9) $$\begin{array}{c} g_{\mathrm{GA}}\left(E\right)=N_{\mathrm{GA}}\exp[-{\left(\frac{E_{\mathrm{GA}}-E}{W_{\mathrm{GA}}}\right)}^{2}] \end{array}$$

(10) $$\begin{array}{c} g_{\mathrm{GD}}\left(E\right)=N_{\mathrm{GD}}\exp[-{\left(\frac{E-E_{\mathrm{GD}}}{W_{\mathrm{GD}}}\right)}^{2}] \end{array}$$

(11) $$\begin{array}{c} D_{\mathrm{it}}\left(E\right)=D_{\mathrm{itA}}\exp\left(\frac{E-E_{C}}{W_{\mathrm{itA}}}\right)+D_{\mathrm{itD}}\exp\left(\frac{E_{V}-E}{W_{\mathrm{itD}}}\right) \end{array}$$

where *g_TD_*(*E*) and *g_TA_*(*E*) denote the density of donor-like tail states and acceptor-like tail states, respectively, while *g_GA_*(*E*) and *g_GD_*(*E*) correspond to Gaussian-distributed acceptor-like and donor-like states. *N_TA_* and *N_TD_* are the effective densities of tail states at the conduction band edge *E_C_* and valence band edge *E_V_*, respectively. *W_TD_* and *W_TA_* represent the characteristic slope energies of the valence band tail and conduction band tail, respectively. For Gaussian defect states, *N_GD_* and *N_GA_* refer to their total densities, *E_GD_* and *E_GA_* are the corresponding peak energies, and *W_GD_* and *W_GA_* denote the characteristic slope energies. *D_itD_* and *D_itA_* stand for the donor-like and acceptor-like interface defect densities, with *W_itD_* and *W_itA_* being their respective slope energies.

**Figure 2:**
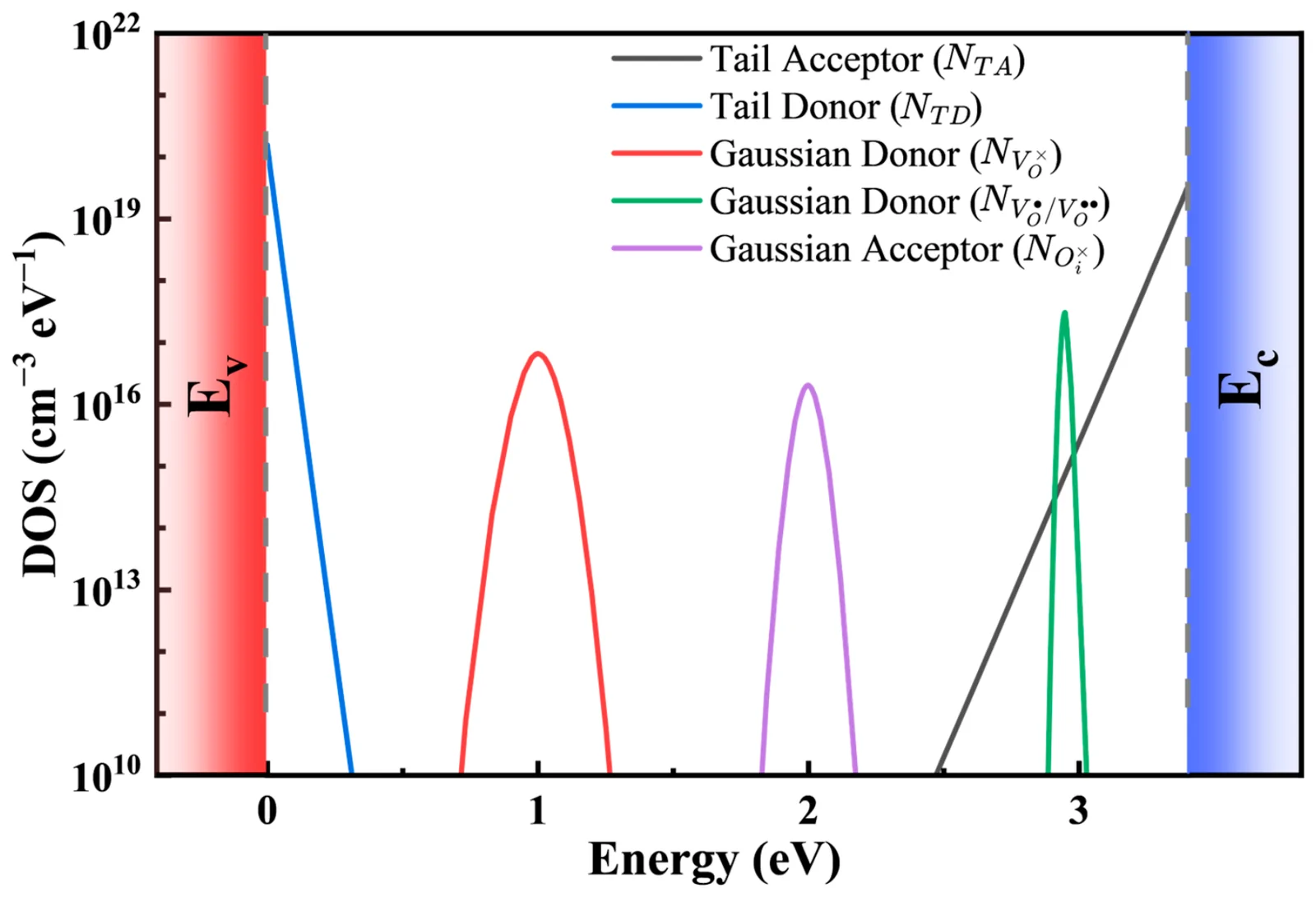
Schematic illustration of the DOS model in the a-IGZTO TFTs.

In this work, donor-like states, acceptor-like states, oxygen vacancies with distinct charge states ($V_{O}^{\times }$,$V_{O}^{\cdot }$/$V_{O}^{\cdot \cdot }$), interstitial oxygen ($O_{i}^{\times }$), and interface defects are incorporated into the simulation [35,36,37]. The corresponding schematic diagram of the density of states (DOS) is illustrated in Figure 2. Because vacuum annealing may change the defect density near the surface of the IGZTO channel, the defect density within the top 15 nm was modeled separately [5,14,18,38,39]. The 15 nm thickness is a literature-informed modeled assumption rather than a directly measured depth in the present IGZTO films. The parameters of the aforementioned defects and the material parameters of IGZTO are summarized in Table 1.

## 3. Results and Discussion

The impact of annealing temperature (25 °C, 150 °C, 250 °C, 350 °C, and 450 °C) on the morphology, structure, and optical transmittance of a-IGZTO thin films and electrical properties of both films and TFTs were studied. The results are given as follows.

### 3.1. Morphology and Structure of IGZTO Thin Films

**Figure 3:**
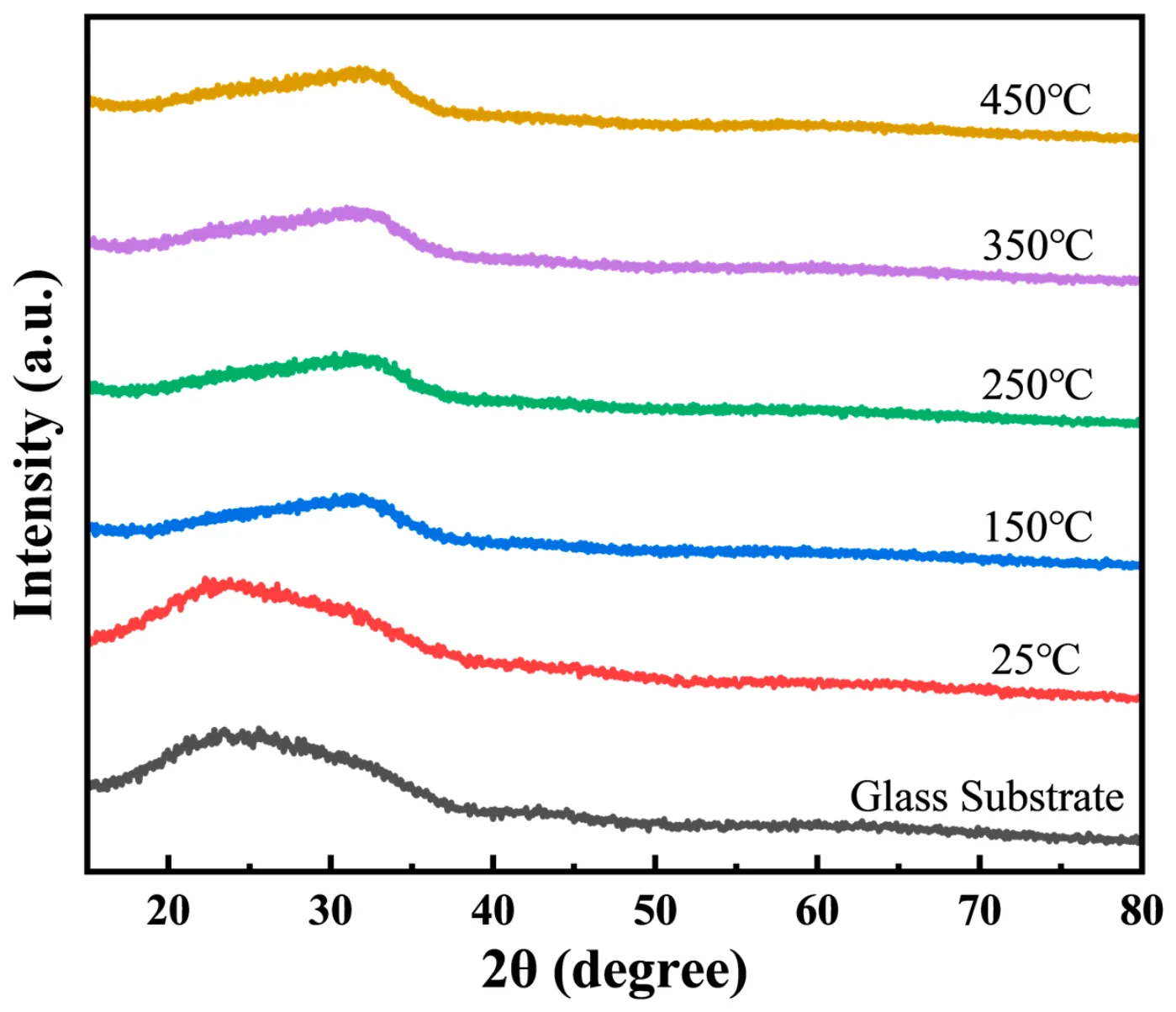
XRD patterns of IGZTO films at different annealing temperatures.

The XRD patterns of both the un-annealed and annealed 50 nm-thick IGZTO thin films are presented in Figure 3. Each pattern contains a single broad halo between 20° and 30° in 2θ, originating from the glass substrate. No characteristic reflection of an IGZTO crystalline phase appears in any sample, so the layers stay amorphous at every annealing temperature. Similar patterns have been observed in our previous works [22,27] and also reported by Y. Li et al., who attributed this result to the synergistic effect of the glass substrate [19].

**Figure 4:**
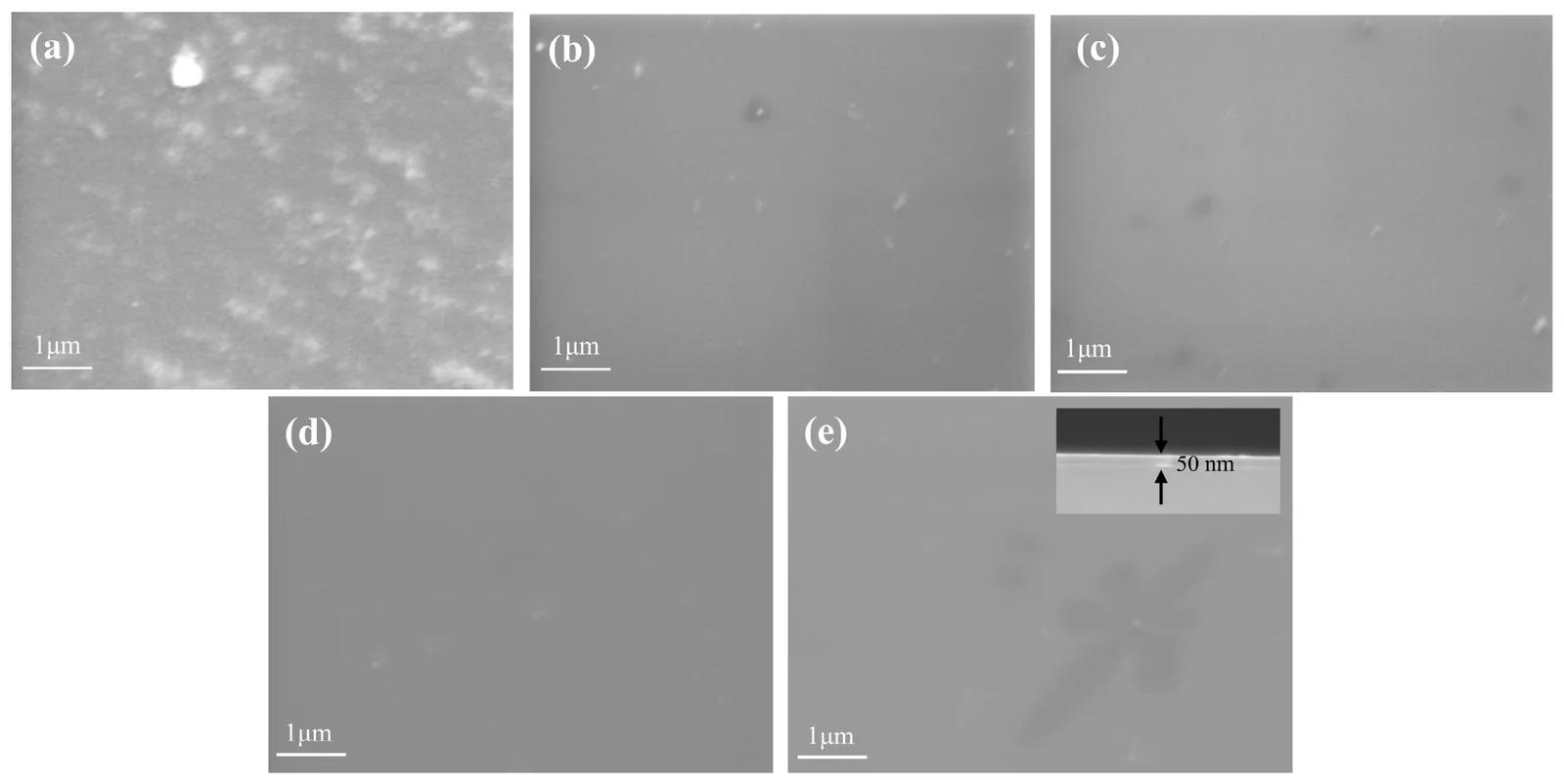
SEM images of IGZTO films at different annealing temperatures: (**a**) 25 °C, (**b**) 150 °C, (**c**) 250 °C, (**d**) 350 °C, and (**e**) 450 °C. The inset in (**e**) shows a cross-sectional image indicating a film thickness of approximately 50 nm.

The SEM measurements were conducted to characterize the surface morphologies of the IGZTO thin films vacuum annealed at different temperatures, with the results shown in Figure 4a–e. The SEM results show that the IGZTO film surfaces become progressively smoother as the vacuum annealing temperature increases from 25 °C to 350 °C, with fewer distinguishable grain-like surface features. This evolution may be explained by thermally activated atomic diffusion and surface rearrangement. At lower annealing temperatures, the limited thermal energy restricts atomic migration, allowing surface irregularities to persist. As the annealing temperature increases toward 350 °C, more atoms can overcome migration barriers, facilitating atomic redistribution and structural relaxation toward a smoother surface [12,42]. At 450 °C, the increase in surface particle size may instead be associated with enhanced surface diffusion that promotes particle coalescence or coarsening [12].

**Figure 5:**
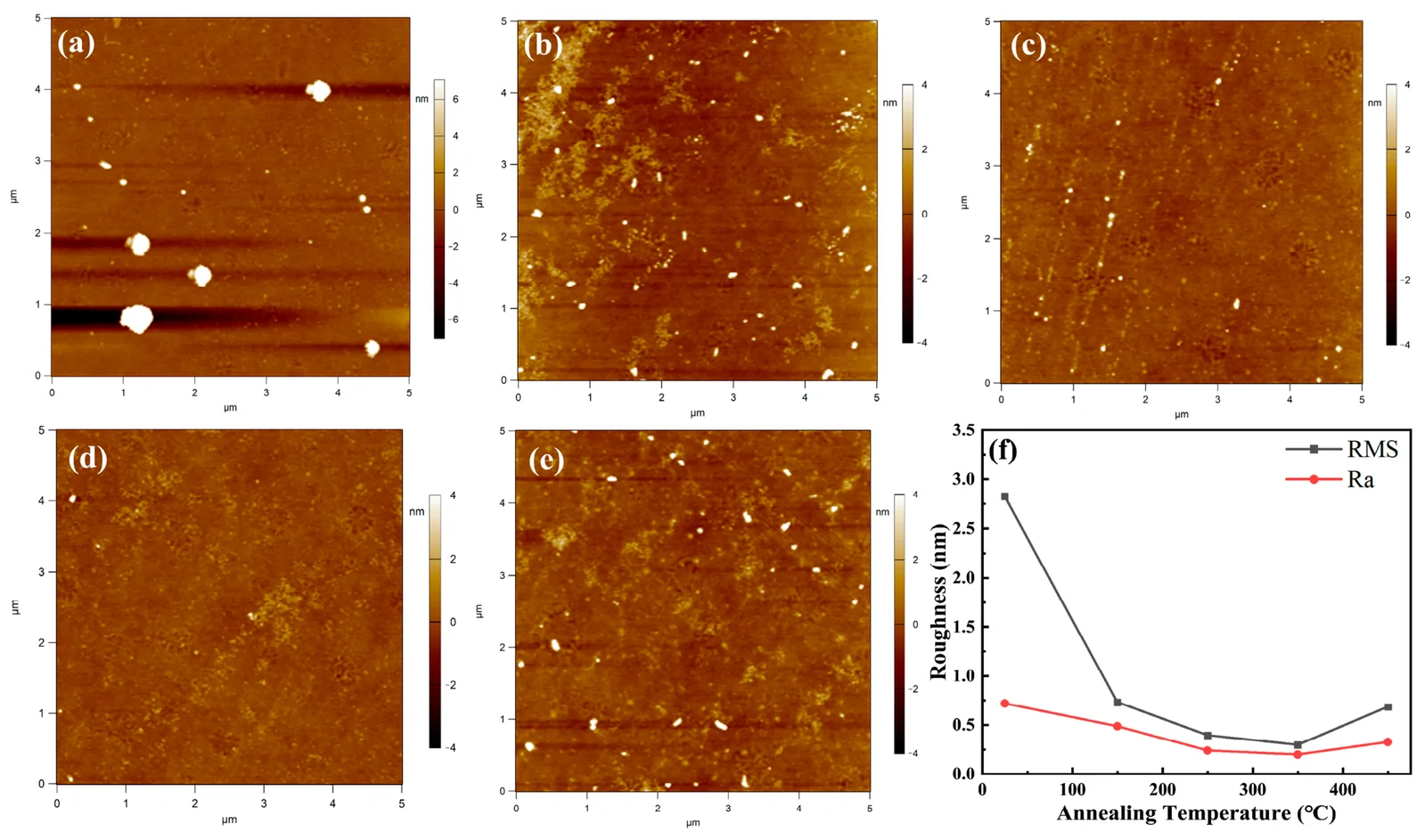
AFM images of IGZTO films at different annealing temperatures: (**a**) 25 °C, (**b**) 150 °C, (**c**) 250 °C, (**d**) 350 °C, (**e**) 450 °C and (**f**) variations of Ra and RMS.

Surface roughness is an important factor for thin-film performance, since it affects carrier transport and scattering [24]. Elevated surface roughness tends to induce carrier scattering and form defect states in the carrier transport channels, ultimately leading to the deterioration of the overall performance of thin films [19]. To determine the surface roughness of IGZTO thin films under different vacuum annealing temperatures, AFM measurements were performed, and the corresponding results are presented in Figure 5a–f. With the increase in annealing temperature, both the root-mean-square (RMS) roughness and arithmetic mean (Ra) roughness of the IGZTO thin films first decrease and then increase, reaching their minimum values of 294 pm (RMS) and 195 pm (Ra) at an annealing temperature of 350 °C. The decrease in RMS and Ra from 25 °C to 350 °C is attributed to the fact that annealing treatment effectively reduces surface particles and defects, thereby lowering the surface roughness of the films. The increase from 350 °C to 450 °C may be associated with enhanced surface atom diffusion, which could promote surface agglomeration and island-like coarsening, thereby increasing surface undulation at higher annealing temperatures [12]. The AFM measurement results are in good agreement with the SEM results presented in Figure 4. A low surface roughness enables more uniform contact between the IGZTO thin film and electrodes, which in turn reduces interfacial scattering and thus enhances the carrier mobility [20]. The vacuum annealing temperature of 350 °C yields the minimum RMS roughness for the IGZTO thin films, which suggests that it is the most favourable condition within this series.

### 3.2. Optical Properties of IGZTO Thin Films

**Figure 6:**
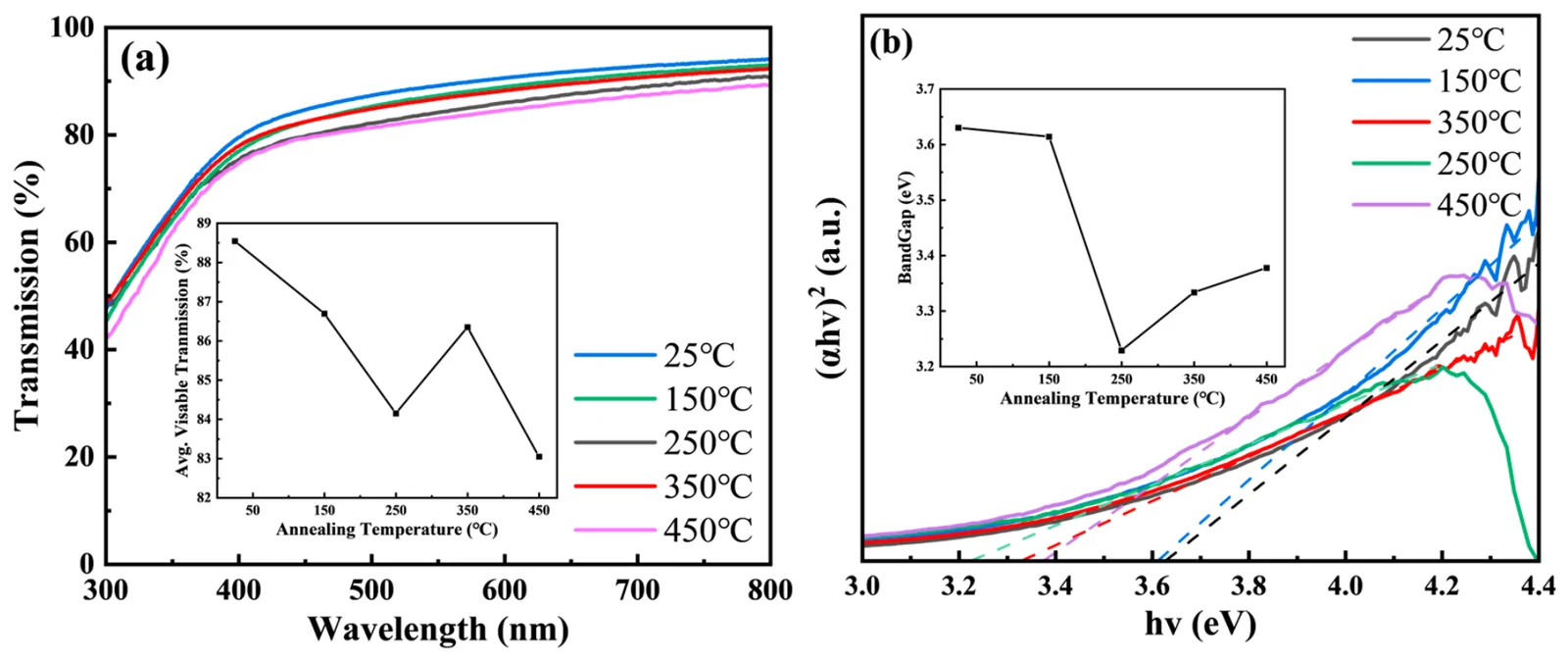
(**a**) Optical transmittance curve of IGZTO films, (**b**) relationship between (*αhv*)^2^ and photon energy *hv*, and optical bandgap width of IGZTO thin films with annealing temperatures.

Figure 6a collects the transmittance spectra of the five films. The average visible light transmittance of the IGZTO thin films exhibited a trend of first decreasing, then increasing and subsequently decreasing with the increase in vacuum annealing temperature, with a maximum value of 88.5% at 25 °C and a minimum value of 83.5% at 450 °C. In the 25–250 °C range, the decrease in visible-light transmittance may be associated with annealing-induced structural relaxation and densification of amorphous IGZTO. Such densification could increase the film density and refractive index, potentially enhancing interfacial reflection and contributing to the observed decrease in transmittance [12]. From 250 °C to 350 °C, thermally activated atomic rearrangement may improve the short-range order of the amorphous network and reduce intrinsic defects through structural relaxation, thereby suppressing defect-related subgap absorption [12,21,42]. When the annealing temperature increases from 350 °C to 450 °C, the enhanced oxygen diffusion and possible oxygen out-diffusion under vacuum may alter the local bonding environment and increase oxygen deficiency. These changes could introduce additional oxygen-vacancy-related gap states and structural disorder, potentially enhancing subgap absorption in the visible region [12,21,42]. The underlying mechanism governing the transmittance evolution is further corroborated by subsequent TCAD simulations.

Figure 6b shows the plots of (*αhv*)^2^ versus *hν* and the variation curve of the *E_g_* with annealing temperature. The *E_g_* of the films first decreases with increasing annealing temperature, reaches the minimum at 250 °C, and then gradually increases from 250 °C to 450 °C, with corresponding values of 3.63, 3.61, 3.22, 3.33 and 3.38 eV, respectively. From 25 °C to 150 °C, the narrowing of the apparent optical band gap originates from the decrease in carrier concentration. At room temperature, the high carrier concentration renders the electron system degenerate, with the Fermi level located inside the conduction band, inducing a pronounced blue shift of the absorption edge and a widened apparent band gap. As the annealing temperature rises to 150 °C, the reduced carrier concentration weakens the degeneracy and attenuates the blue shift, leading to a relative red shift of the absorption edge and a narrowed apparent optical band gap. In the range of 350–450 °C, the rising carrier concentration intensifies the electron degeneracy, resulting in a blue-shifted absorption edge and a widened apparent optical band gap. This phenomenon is known as the Burstein–Moss effect. For the as-deposited direct band gap IGZTO thin films before annealing, their intrinsic band gap *E_go_* can be expressed as [2,42]

(12) $$\begin{array}{c} E_{\mathrm{go}}=E_{c}^{o}\left(K_{F}\right)-E_{v}^{o}\left(K_{F}\right) \end{array}$$

where $E_{c}^{o}\left(K_{F}\right)$ and $E_{v}^{o}\left(K_{F}\right)$ denote the unperturbed initial and final states, respectively, and *K_F_* represents the Fermi wave vector.

After annealing treatment, the band gap broadening $∆E_{g}^{\mathrm{BM}}$ induced by the Burstein–Moss shift can be expressed as

(13) $$\begin{array}{c} ∆E_{g}^{\mathrm{BM}}=\frac{\hbar^{2}}{2m_{\mathrm{cv}}^{*}}{\left(3\pi^{2}n_{e}\right)}^{\frac{2}{3}} \end{array}$$

where *n_e_* is the carrier concentration, $m_{\mathrm{cv}}^{*}$ represents the reduced effective mass associated with optical transitions between the valence and conduction bands. It shows that the Burstein–Moss shift is proportional to $n_{e}^{2/3}$ and inversely proportional to the reduced effective mass $m_{\mathrm{cv}}^{*}$, which can be obtained from the following equation:

(14) $$\begin{array}{c} \frac{1}{m_{\mathrm{cv}}^{*}}=\left[\frac{1}{m_{c}^{*}}+\frac{1}{m_{v}^{*}}\right] \end{array}$$

where $m_{c}^{*}$ and $m_{v}^{*}$ denote the electron effective mass near the conduction-band minimum and the hole effective mass near the valence-band maximum, respectively.

Based on the analysis of the aforementioned equations, it can be concluded that the decrease in carrier concentration of the films during annealing at 25–150 °C leads to a weakened band gap broadening, thus resulting in a decrease in the optical band gap *E_g_*. On the other hand, residual stress may occur in the films during the deposition process. Its partial relaxation during low-temperature annealing could modify local inter-atomic distances and the consequent electronic band structure, potentially contributing to the *E_g_* decrease as the annealing temperature increases to 250 °C [43,44]. When the annealing temperature is increased to 450 °C, the IGZTO films increase in $V_{O}^{\times }$ density, enhancing the Burstein–Moss effect, and the superimposed changes in structural disorder act together—all of which contribute to the broadening of *E_g_*. In addition, the annealing at 450 °C induces an increase in oxygen vacancies within the films, which elevates the carrier concentration and thus intensifies the band gap broadening effect; this further enhances the Burstein–Moss effect [27,41,42], leading to a subsequent increase in *E_g_*.

**Figure 7:**
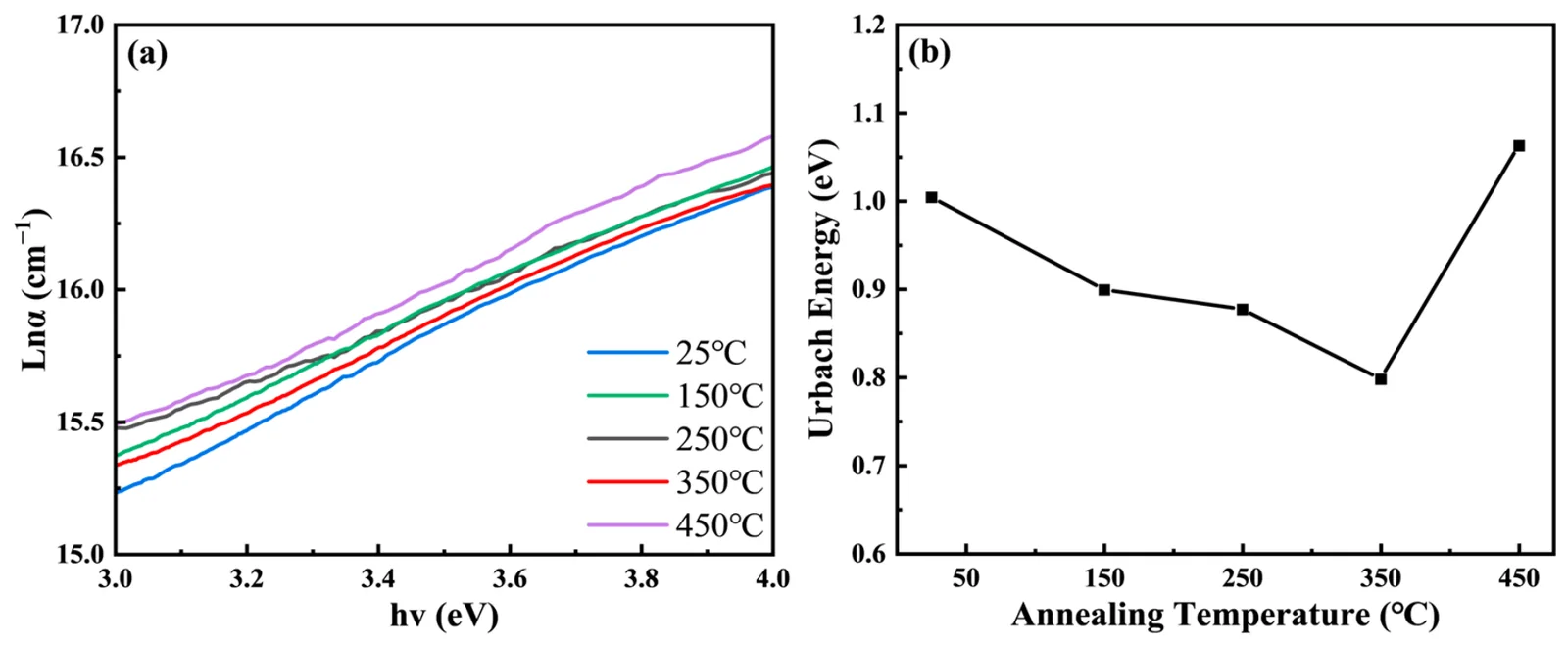
(**a**) Plots of *lnα*-*hυ* and (**b**) *E_U_* of IGZTO films at different annealing temperatures.

The *lnα*-*hυ* curves, together with the *E_U_* values derived from them, are collected in Figure 7 for the five films. It shows that the *E_U_* of the IGZTO thin films first decreases and then increases with increasing vacuum annealing temperature, reaching a minimum of 0.80 eV at 350 °C and a maximum of 1.06 eV at 450 °C. A larger *E_U_* generally suggests greater structural disorder and more pronounced defect-related band-tail states [33]. The decrease in *E_U_* from 25 °C to 350 °C may be associated with thermally activated atomic rearrangement and structural relaxation. These processes could improve the local ordering of the amorphous network and reduce disorder-related localized states, thereby narrowing the absorption tail and lowering *E_U_* [21]. Compared with the film annealed at 350 °C, the higher *E_U_* observed at 450 °C may be partly associated with enhanced oxygen out-diffusion under vacuum and the resulting increase in oxygen deficiency [16,33]. Changes in local coordination and oxygen-vacancy-related electronic states could contribute to additional structural disorder and a broader absorption tail.

### 3.3. Electrical Properties of Annealed IGZTO Thin Films

**Figure 8:**
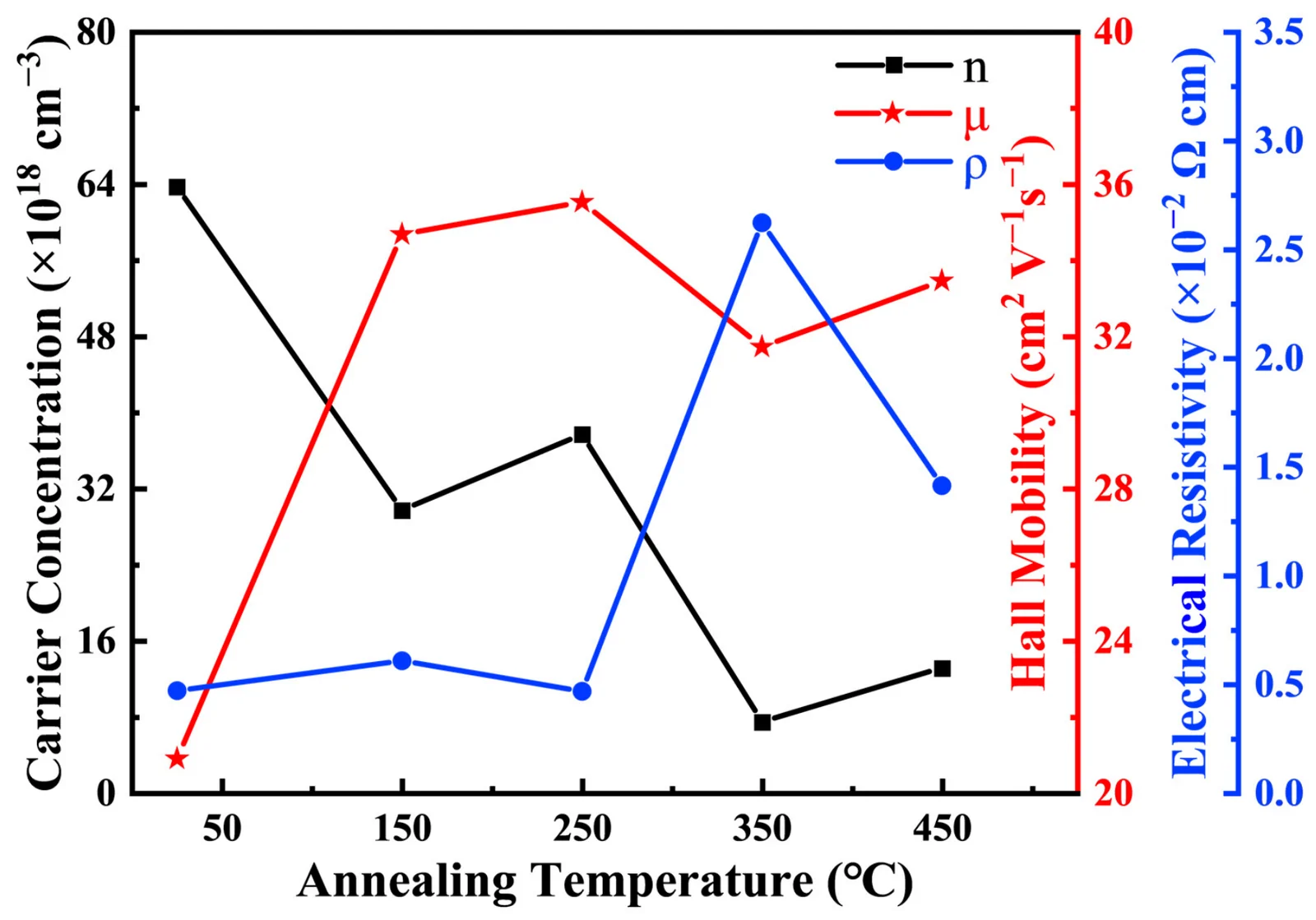
Annealing-temperature dependence of the carrier concentration, Hall mobility and resistivity of the IGZTO layers.

Hall measurements were used to follow the transport behaviour of the annealed layers; the data are collected in Figure 8 and Table 2. All samples were n-type. The free-carrier concentration did not vary monotonically; it started at 6.37 × 10^19^ cm^−3^ for the as-deposited film, fell to 2.97 × 10^19^ cm^−3^ after annealing at 150 °C, rose again to 3.77 × 10^19^ cm^−3^ at 250 °C, dropped sharply to 7.44 × 10^18^ cm^−3^ at 350 °C, and finally recovered to 1.31 × 10^19^ cm^−3^ at 450 °C.

The as-deposited film had a Hall mobility of 20.89 cm^2^ V^−1^ s^−1^. Annealing raised it into a narrow 31–36 cm^2^ V^−1^ s^−1^ band, i.e., by roughly 50%, with the maximum of 35.53 cm^2^ V^−1^ s^−1^ recorded at 250 °C and 31.72 cm^2^ V^−1^ s^−1^ at 350 °C.

Transport in the channel of an amorphous oxide is commonly described by percolation through a potential landscape [2], so that the Hall mobility is expected to grow with the carrier concentration; the measured mobility therefore reflects how efficiently percolation paths are established at a given carrier density. The carrier density itself is set by the $V_{O}^{\times }$ population of the layer, which is examined in Section 3.4 by XPS. The as-deposited film illustrates the first point well; it contains the largest number of carriers yet shows the lowest mobility, because its highly disordered network offers few connected percolation paths.

Table 3 places the 350 °C vacuum-annealed film of this study alongside previously reported IGZO, ITZO and IGZTO layers. The Hall mobility obtained here is higher than the values published for IGZO and ITZO, although it remains below the 65 cm^2^ V^−1^ s^−1^ reported for a sol–gel derived IGZTO film [31]; solution processing, however, is less convenient for device integration. The present 31.72–35.53 cm^2^ V^−1^ s^−1^ range is close to the 35.68 cm^2^ V^−1^ s^−1^ that we obtained earlier for an IGZTO film annealed in air at 150 °C [22], but that film also carries 3.06 × 10^19^ cm^−3^ free electrons, which is higher than desirable for a transistor channel that should remain fully depleted in the off state.

### 3.4. Composition and Chemical Configuration of IGZTO Thin Films

**Figure 9:**
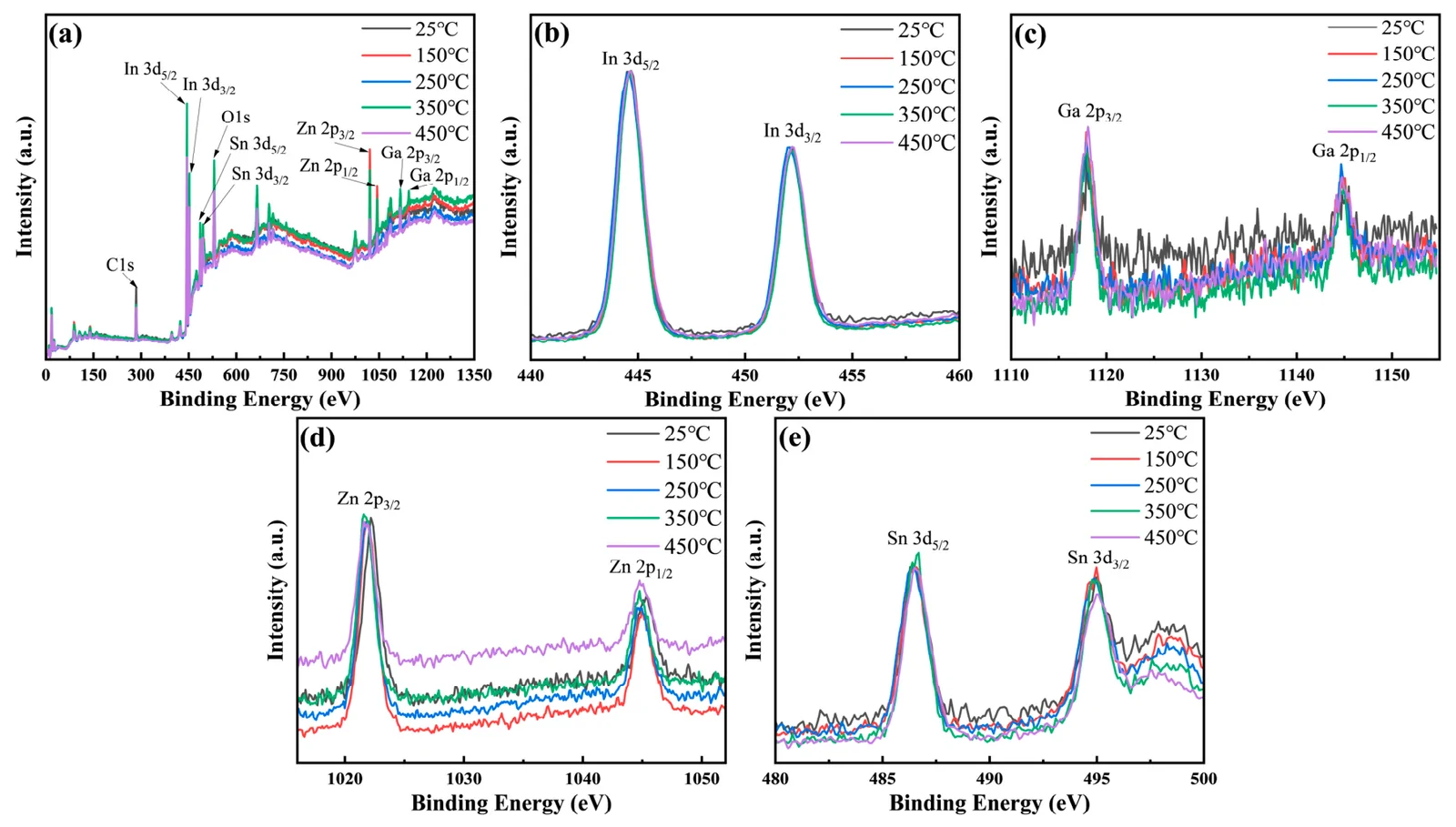
(**a**) Total XPS spectra and (**b**–**e**) high-resolution spectra of In 3d, Ga 2p, Zn 2p, Sn 3d XPS of IGZTO films at different annealing temperatures.

XPS was used to determine the elemental composition and the oxidation states of the annealed layers. Figure 9a shows the survey spectra and Figure 9b–e display the high-resolution regions of In 3d, Ga 2p, Zn 2p and Sn 3d; the atomic percentages obtained from the integrated peak areas are listed in Table 4. Indium appears exclusively in the In^3+^ state, with the 3d_5_/_2_ and 3d_3_/_2_ doublet at 444.6 and 452.2 eV. The Ga 2p_3_/_2_ and Ga 2p_1_/_2_ lines are centred at about 1118 and 1146 eV, consistent with Ga^3+^, and the Sn 3d_5_/_2_ and 3d_3_/_2_ components at approximately 486.5 and 495.4 eV identify tin as Sn^4+^.

**Figure 10:**
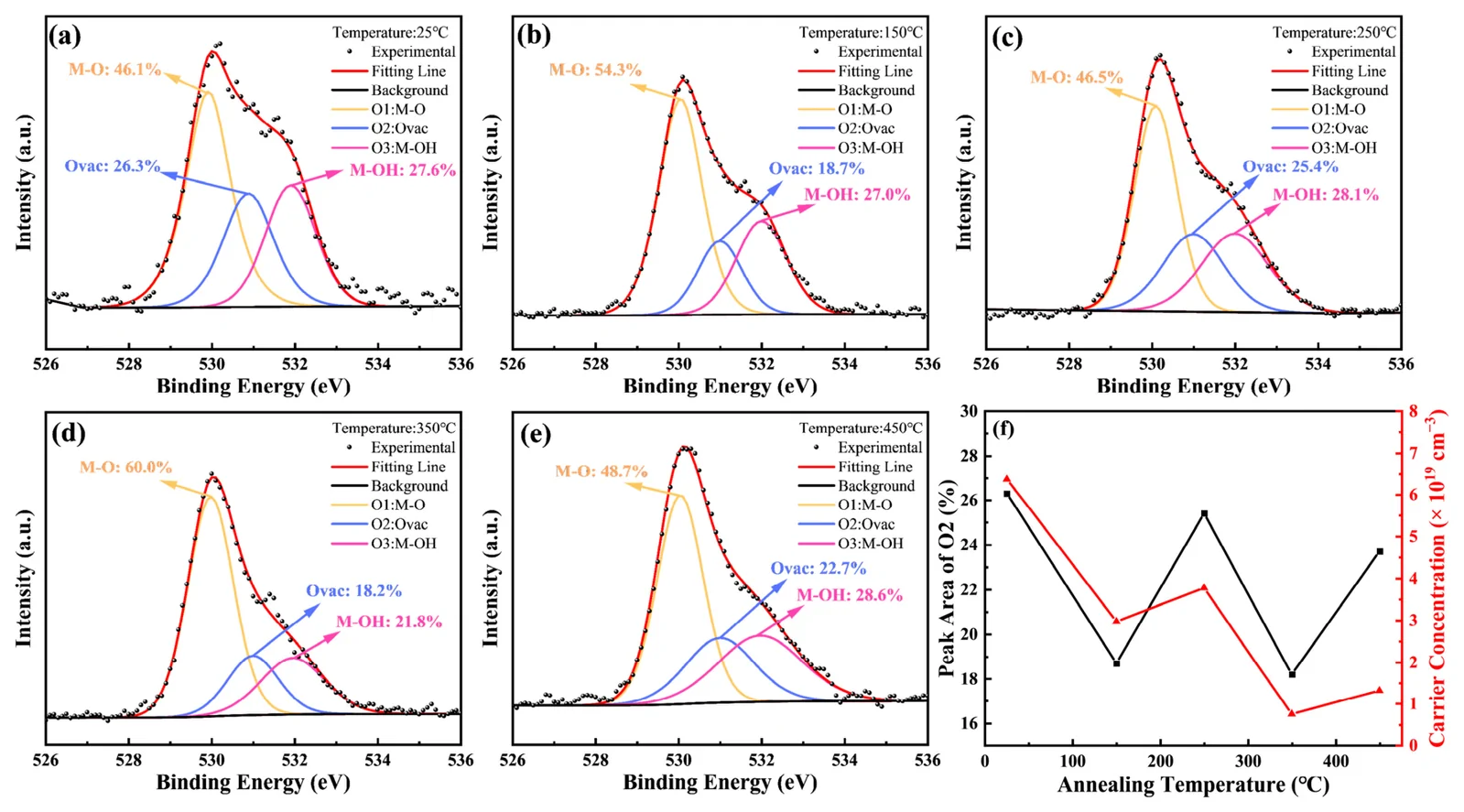
O 1s XPS spectra of the IGZTO films annealed at (**a**) 25 °C, (**b**) 150 °C, (**c**) 250 °C, (**d**) 350 °C and (**e**) 450 °C, together with (**f**) the annealing-temperature dependence of the O2 peak fraction and of the carrier concentration.

The O 1s region of the five samples is resolved in Figure 10a–e. Three Gaussian–Lorentzian components reproduce every spectrum: O1 near 530.0 eV, O2 near 531.0 eV and O3 near 532.0 eV, with fitting uncertainties of 0.2, 0.1 and 0.3 eV, respectively. O1 represents oxygen that is fully coordinated by metal cations (Zn–O, In–O) inside the oxide network [5,18,46,47], O2 corresponds to oxygen in the incompletely coordinated regions of the matrix and therefore tracks the $V_{O}^{\times }$ population [48], and O3 arises from species adsorbed at the surface, such as O_2_ and H_2_O [5]. Deconvolution cannot give an absolute $V_{O}^{\times }$ count, but because the same fitting constraints were applied to every sample, the relative areas remain comparable; the same procedure has been used by Kim et al. to follow oxygen vacancies in TFTs [48]. The O2 share does not vary monotonically along the series. It passes through 26.3%, 18.7%, 25.4%, 18.2% and 22.7% as the annealing temperature rises from 25 to 450 °C.

Relaxation of the network accounts for the drop in the $V_{O}^{\times }$ density between 25 and 150 °C, since oxygen atoms can refill the vacancies during this stage. Between 150 and 250 °C the trend reverses: weakly bound oxygen becomes mobile and escapes from the interior into the evacuated ambient, and because a higher temperature accelerates this loss, both the vacancy population and the amount of oxygen adsorbed at the surface grow, so that the $V_{O}^{\times }$ concentration rises. From 250 to 350 °C the thermal energy is again sufficient for relaxation to dominate—interstitial oxygen migrates back to the vacancies and the network re-arranges so that fewer under-coordinated cations remain—and consequently both the $V_{O}^{\times }$ density and the free-carrier concentration fall. A further rise of the O2 fraction is observed at 450 °C because the comparatively weak Zn–O and In–O bonds allow oxygen to leave the layer through a defect-assisted kick-out process [10], which increases the $V_{O}^{\times }$ density. Table 2 shows that this high-temperature step is accompanied by scission of Zn–O and In–O bonds and by appreciable loss of Zn, so that the relative atomic fractions of In, Ga and Sn rise [49]; the extensive breaking of Zn–O bonds may be a major reason for the strong increase in $V_{O}^{\times }$. The close correspondence between the $V_{O}^{\times }$ concentration deduced from the O2 fraction and the carrier density plotted in Figure 10f mirrors the Hall data and supports the interpretation of the XPS results.

### 3.5. Electrical Performance of IGZTO TFTs

**Figure 11:**
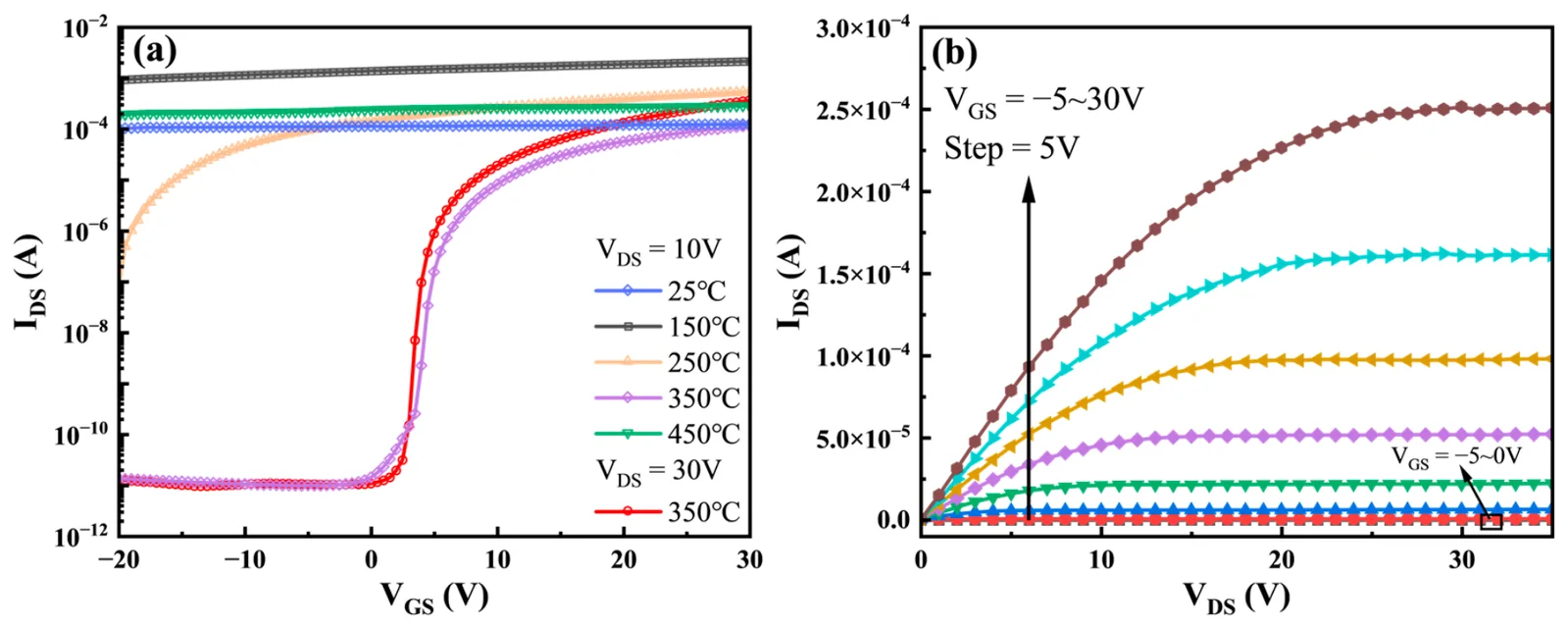
(**a**) Transfer curves measured on IGZTO TFTs subjected to the different vacuum anneals. (**b**) *I*_DS_–*V*_DS_ output family of the 350 °C device recorded at different gate voltages.

Figure 11a compares the transfer curves recorded after each anneal. Only the 350 °C device exhibits a well-defined on/off transition; the samples treated at 25, 150, 250 and 450 °C remain conductive over the whole gate-voltage range and therefore show no usable switching. The reasons differ as follows: at 25 and 150 °C, relaxation of the amorphous network is incomplete; at 250 °C, the channel contains a large number of $V_{O}^{\cdot }$/$V_{O}^{\cdot \cdot }$ donor-like states that supply far more carriers than can be depleted; and at 450 °C, the network has become excessively disordered and oxygen deficient [14,50]. Because only the 350 °C device could be operated in saturation, its figures of merit were extracted at *V*_DS_ = 30 V, giving *μ*_Sat_ = 19.7 cm^2^ V^−1^ s^−1^, *V*_TH_ = 4.72 V, *SS* = 0.56 V dec^−1^ and *I*_on_/*I*_off_ = 8.1 × 10^7^. Vacuum annealing at 350 °C therefore appears to be the best condition in this series.

The output characteristics of the 350 °C device are shown in Figure 11b for gate voltages from −5 to 30 V in steps of 5 V. The drain current scales with *V*_GS_, which is consistent with gate control of the channel conductance. Each curve rises almost linearly at small *V*_DS_, bends over and then flattens into saturation, and no marked current crowding appears near the origin, which indicates that the Al/IGZTO interfaces are reasonably Ohmic contacts. The device thus behaves as a well-formed field-effect transistor after annealing at 350 °C.

To assess device-to-device reproducibility, four additional IGZTO TFTs annealed at 350 °C were characterized. Using a consistent fitting range, the key extracted performance parameters are as follows: *μ_Sat_* = 19.64 ± 2.13 cm^2^ V^−1^ s^−1^, *V_TH_* = 4.89 ± 0.95 V, *SS* = 0.55 ± 0.11 V dec^−1^ and *I_on_/I_off_* = (4.75 ± 4.18) × 10^7^ (mean ± standard deviation, n = 4). These devices consistently exhibited switching behaviour, with relatively small variation in mobility. However, the *I_on_/I_off_* varied substantially, and successive sweeps showed shifts in the transfer characteristics, indicating that it requires to further control off-state behaviour and adjust measurement-history dependence.

**Figure 12:**
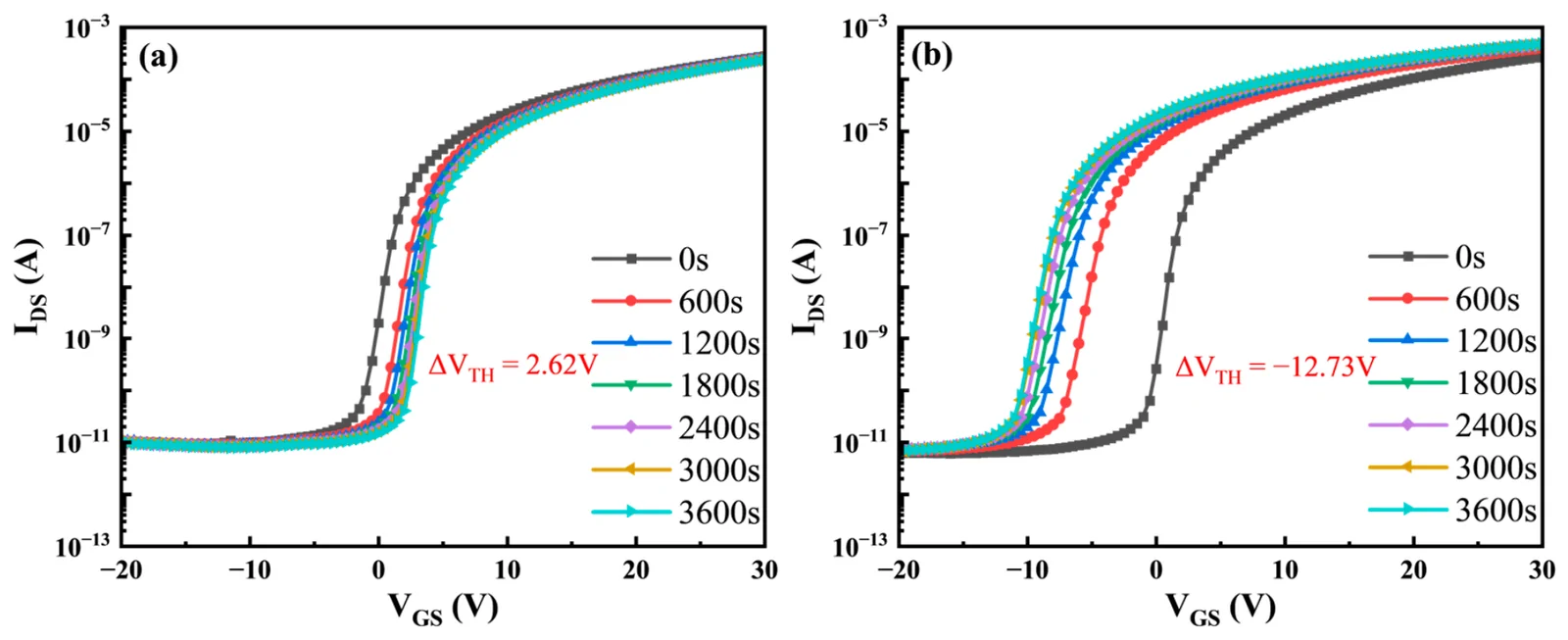
Evolution of the transfer characteristics of the IGZTO TFT annealed in vacuum at 350 °C under (**a**) positive bias stress (PBS, V_GS_ = +20 V) and (**b**) negative bias stress (NBS, V_GS_ = −20 V), with stress duration ranging from 0 to 3600 s.

The bias-stress stability of the IGZTO TFT vacuum annealed at 350 °C was further evaluated under PBS and NBS [8], which are shown in Figure 12a and Figure 12b, respectively. As the stress duration increases from 0 to 3600 s, the transfer curves gradually shift toward more positive gate voltages under PBS, with a *V_TH_* shift of +2.62 V after 3600 s. The *I_off_* remained approximately at the 10^−11^ A level, while the curves in the high-gate-voltage region showed relatively small changes. In contrast, NBS induced a substantially larger negative shift, reaching −12.73 V after 3600 s, together with an increase in drain current at a given gate voltage. Under both stress polarities, the additional curve shifts became smaller at longer stress duration.

The positive shift under PBS may be associated with electron trapping at or near the IGZTO/SiO_2_ interface and within the gate dielectric. The resulting negative trapped charge would require a more positive gate voltage to establish a comparable channel current [51]. The larger negative shift under NBS may involve changes in the occupation and charge states of oxygen-related defects, potentially accompanied by electron release and the formation of positively charged donor states [50]. Notably, Choi et al. also reported better PBS stability than NBS stability in IGZTO TFTs and attributed this asymmetry to the distributions of shallow- and deep-level defects [18]. The accompanying change in the *SS* suggests that the NBS response may involve defect-state evolution in addition to an electrostatic shift. These results indicate that the optimized vacuum annealing yields functional switching characteristics, while further control of interface and oxygen-related defects is needed to improve NBS stability.

Table 5 presents the performance of the present 350 °C device together with values reported for representative IGZO and IGZTO TFTs. Most IGZTO devices [25,51,52] outperform their IGZO counterparts [50,53], which is consistent with the benefit of Sn incorporation, and among the IGZTO devices those deposited by magnetron sputtering [22,51,52] show higher mobilities than sol–gel derived ones [25]. Within the sputtered group, our transistor offers a competitive mobility and a satisfactory *I*_on_/*I*_off_ of 8.1 × 10^7^. The largest mobility in the table, 40.4 cm^2^ V^−1^ s^−1^, belongs to a bilayer IGZTO channel with graded oxygen doping [47]. Because the device architecture and the parameter-extraction procedure differ from report to report, such a comparison can only be indicative; ALD growth or a dual-channel design could reasonably be expected to improve the present device further. Stability data are not compared here, since the stress conditions used in the literature vary too widely to be placed on a common footing.

### 3.6. TCAD Simulation Results of IGZTO TFTs

**Figure 13:**
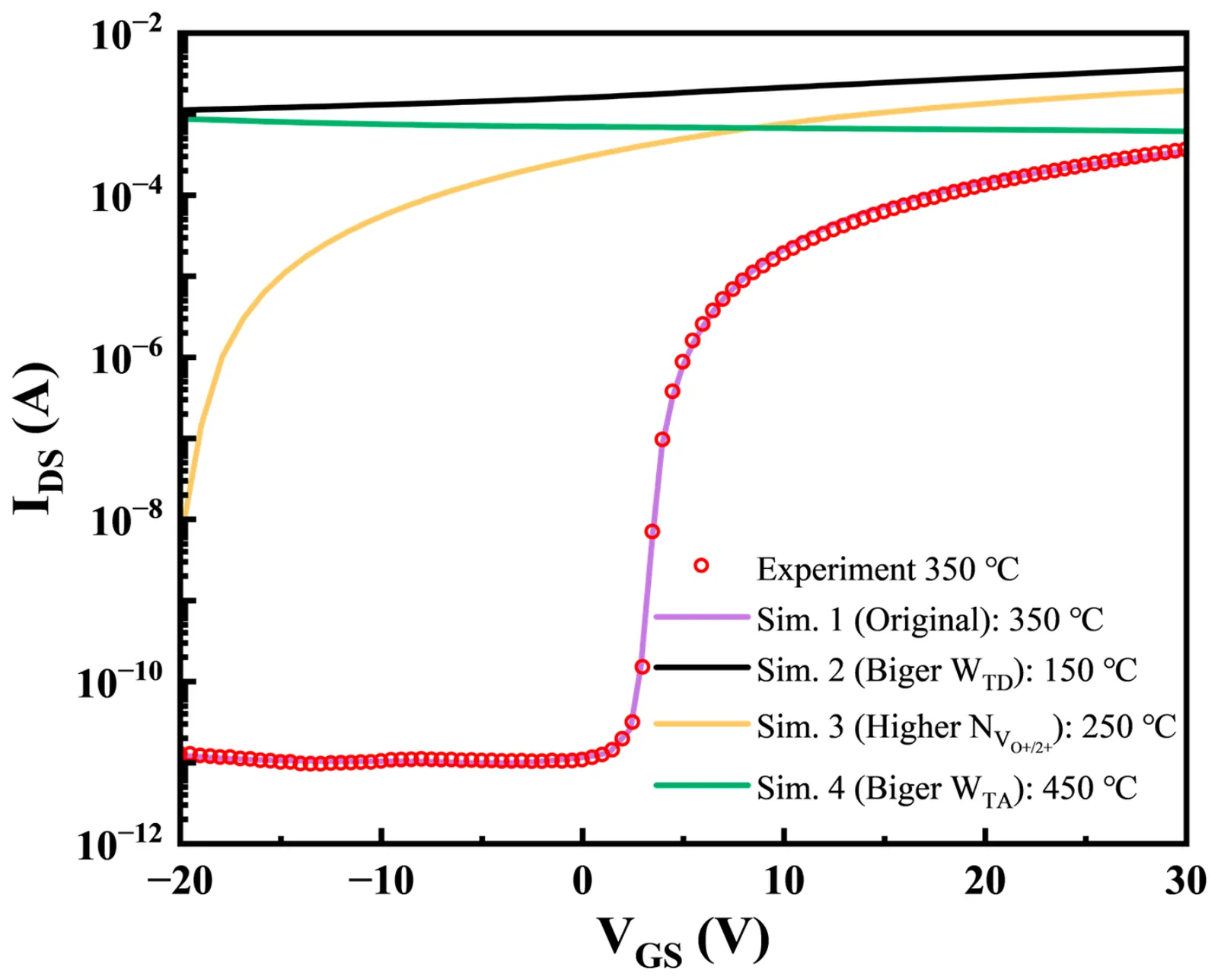
The comparison of the transfer curves simulated by TCAD (lines) and the experiment one of 350 °C annealed (symbols) a-IGZTO TFTs.

To gain deeper insight into the effects of vacuum annealing on defect states and carrier transport in IGZTO TFTs, TCAD simulations were performed. The arguments of the DOS model and basic electronic parameters for the IGZTO TFT are listed in Table 1, and the corresponding calculation is called Simulation 1. A comparison between the experimental and simulated results is presented in Figure 13. It shows that the curve of Simulation 1 agrees reasonably well with the experimental one of the IGZTO TFT subjected to vacuum annealing at 350 °C, which indicates that the TCAD model reproduces the main features of the measured device.

The influence of different vacuum annealing temperatures on the structural disorder and $V_{O}^{\times }$ density of IGZTO TFTs was analyzed in Section 3.2 and Section 3.4. From combining with the *E_U_* and XPS characterization results, we can find that the sample of 350 °C has both the smallest structural disorder and $V_{O}^{\times }$ density, since it has the minimum *E_U_* (Figure 7b) and the lowest O2 peak area percentage and carrier concentration (Figure 10d). This suggests that the IGZTO TFTs treated under 150 °C, 250 °C and 450 °C annealing conditions have larger structural disorder and higher $V_{O}^{\times }$ density than the one at 350 °C.

The structural disorder can be expressed by *W_TA_*, the characteristic slope energy of the conduction band tail (CBT), or *W_TD_*, that of the valence band tail (VBT) [28,29], respectively. In order to reflect the effect of the large structural disorder and high $V_{O}^{\times }$ density on the TFT performance, the values of *W_TD,_ W_TA_,* and $N_{V_{O}^{\cdot }/V_{O}^{\cdot \cdot }}$ were, respectively, increased, and three other simulations were carried out. Specifically, for each of these three simulations, only one parameter (*W_TD,_ W_TA_,* or $N_{V_{O}^{\cdot }/V_{O}^{\cdot \cdot }}$) deviates from the original value set in Table 1, while all other parameters remain unchanged. In detail, the separately tuned parameter of each simulation is shown below: *W_TD_* was increased from 0.124 eV to 0.9 eV (Simulation 2), the $N_{V_{O}^{\cdot }/V_{O}^{\cdot \cdot }}$ was enhanced from 7 × 10^17^ cm^−3^ to 5 × 10^18^ cm^−3^ (Simulation 3), and *W_TA_* was raised from 0.04 eV to 2.5 eV (Simulation 4).

Since XPS analysis provides only relative information about oxygen-vacancy-related components, it is difficult to determine their absolute concentrations. Furthermore, although the *E_U_* can reflect the degree of structural disorder and defect-related band-tail states, defects concentrated within a limited region, such as the near-surface layer considered in this study, may also appreciably increase the measured *E_U_* value. Therefore, *E_U_* alone cannot be used to directly determine the specific defect density or its distribution within the bulk of the film. Therefore, these three supplementary simulations are intended primarily for qualitative elucidation of the underlying mechanisms; thus, no detailed quantitative fitting is performed.

The results of Simulations 2, 3 and 4 are exhibited in Figure 13, in which no obvious switching characteristics were observed, and they are very close to the transfer curves of a-IGZTO TFTs vacuum annealed at 150 °C, 250 °C and 450 °C, respectively. These results can be explained as follows: At a vacuum annealing temperature of 150 °C, insufficient structural relaxation leads to a large *W_TD_*, resulting in the absence of distinct switching characteristics (curve of Simulation 2). At 250 °C, sufficient structural relaxation reduces *W_TD_*; however, inadequate oxygen diffusion and redistribution give rise to a high $V_{O}^{\times }$ density, which also yields no clear switching behaviour (curve of Simulation 3). At 450 °C, excessive oxygen diffusion and desorption, coupled with partial structural degradation induced by the elevated temperature, lead to a reduced *W_TD_*, but an increased *W_TA_*, consequently failing to exhibit distinct switching characteristics (curve of Simulation 4).

**Figure 14:**
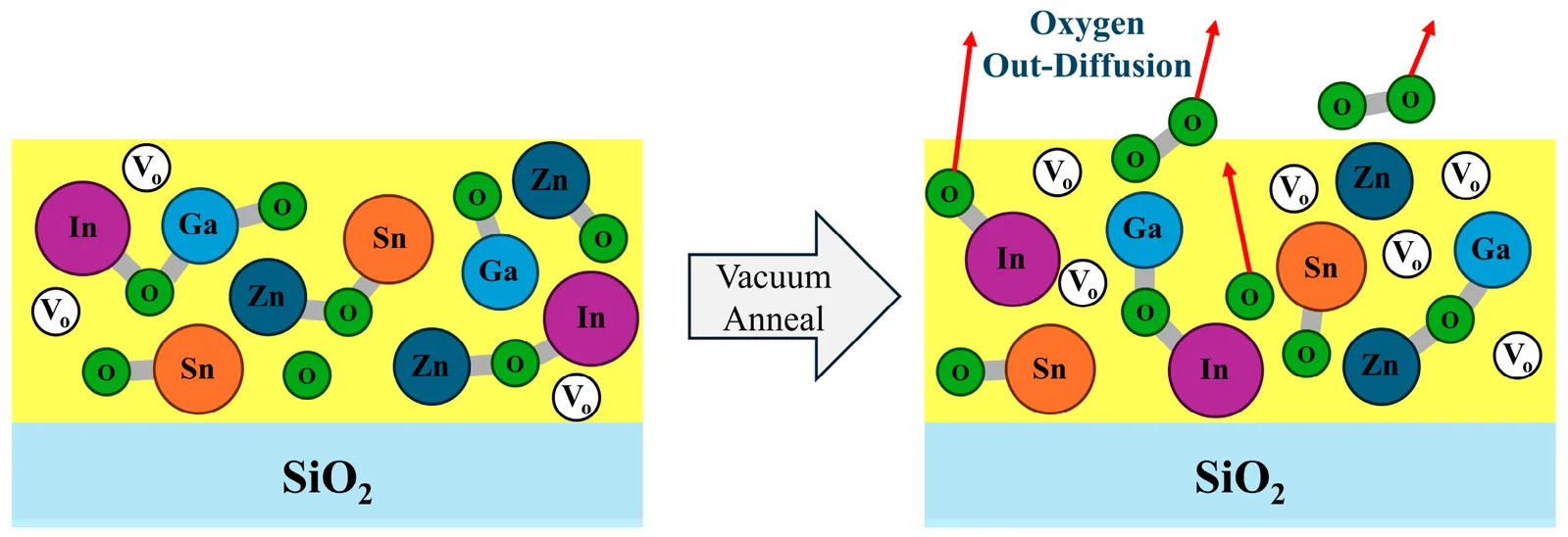
Schematic illustration of oxygen diffusion in IGZTO thin films before and after vacuum annealing.

After vacuum annealing, the $V_{O}^{\times }$ density at the IGZTO surface is likely to be higher than that in the bulk, which stems from the combined effect of oxygen diffusion toward the surface driven by the external high-vacuum environment and oxygen desorption from the surface [14,18,54], as illustrated in Figure 14.

Due to the involvement of oxygen diffusion and desorption during the treatment process, vacuum annealing has been reported to induce a larger increase in $V_{O}^{\times }$ density and exert a more pronounced impact on the structural disorder of the films than air annealing [28,29,40,41,45], so the precise control of the vacuum annealing temperature is very critical. Identifying the optimal vacuum annealing temperature therefore remains an important issue for improving the performance of IGZTO films and TFTs.

To further evaluate the extent to which structural defects concentrated in the surface region affect the overall electrical performance of the device, simulations were performed by varying *W_TA_* and *W_TD_*. Specifically, two scenarios were considered: in the first situation (Situation 1), *W_TA_* and *W_TD_* were varied throughout the entire channel layer, whereas in the second situation (Situation 2), they were varied only within the top 15 nm surface region of the IGZTO channel; other conditions are the same as those in Table 1.

**Figure 15:**
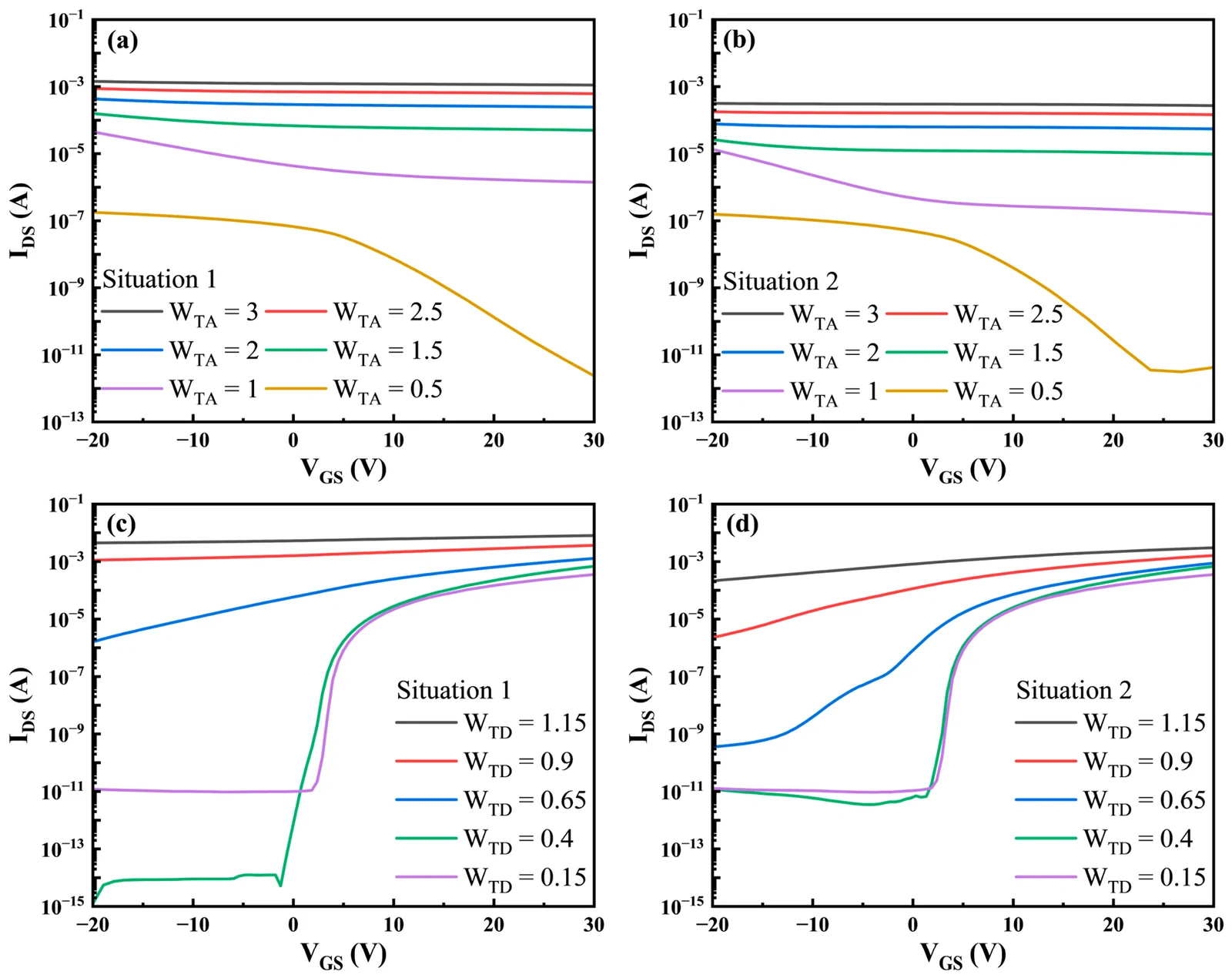
Sensitivity of the simulated IGZTO TFT transfer characteristics to the characteristic energies of acceptor-like and donor-like tail states. (**a**,**b**) Variation in *W_TA_* and (**c**,**d**) variation in *W_TD_*, with the parameter changes applied to (**a**,**c**) the entire 50 nm channel (Situation 1) and (**b**,**d**) the top 15 nm surface region (Situation 2).

Figure 15 compares the effects of varying *W_TA_* and *W_TD_* within the surface region and throughout the channel. In Situation 1, increasing *W_TA_* raises the simulated current and progressively weakens its gate-voltage dependence. In Situation 2, a similar increase in current is observed as *W_TA_* increases from 0.5 to 3 eV. Increasing *W_TD_* generally raises the low-gate-voltage current and suppresses the switching contrast in both situations. The curves retain pronounced switching behaviour at *W_TD_* = 0.15 and 0.4 eV, but become increasingly difficult to turn off at larger values. The different responses of the two situations indicate that device behaviour depends on both the energy distribution and spatial location of the tail states.

These responses may arise from changes in localized-state occupation and the resulting electrostatic potential within the channel. Previous TCAD studies on a-IGZO TFTs have linked the changes in subgap states, including tail-distributed acceptor-like defects, to the variations in transfer characteristics and subthreshold behaviour [28]. Broader tail-state distributions may therefore modify carrier trapping and charge compensation, thereby altering gate control. Moreover, the studies on a-ITGZO TFTs have shown that the influence of defects depends on their spatial relationship to the conducting channel [52]. By analogy, the present simulations suggest that the disorder confined to a surface region could substantially affect the overall device response, without requiring an identical change throughout the film. However, this sensitivity analysis does not establish the actual depth of the disordered region or uniquely determine the defect distribution. Although Urbach tails have been associated with structural disorder [33], the measured optical Urbach energy should not be directly equated with either *W_TA_* or *W_TD_*.

The above results suggest that vacuum annealing at an appropriate temperature can improve the performance of IGZTO TFTs. However, the optimal annealing condition identified in the present study was obtained using bottom-gate staggered a-IGZTO TFTs and therefore cannot be directly extended to top-gate devices. Differences in device architecture and in the deposition sequence of the gate insulator (GI) and channel layer can produce distinct interface properties and consequently different transfer characteristics [52]. If vacuum annealing is performed before GI deposition in a top-gate TFT, appropriate annealing may promote structural relaxation and improve the surface smoothness of the channel layer, potentially facilitating the subsequent formation of a high-quality channel/GI interface [50,53]. However, vacuum annealing may also induce excessive oxygen loss and increase the concentration of oxygen vacancies and free carriers, making the channel difficult to deplete, increasing the off-state current, and shifting the threshold voltage negatively [16,18,35]. Moreover, annealing performed before GI deposition mainly affects the exposed channel layer, whereas annealing performed after GI deposition acts on both the channel and GI layers. The dielectric overlay may restrict oxygen exchange and introduce additional interface diffusion, charge-trapping, and defect-related processes [50,53]. Therefore, the optimal annealing conditions determined for the bottom-gate devices in this study cannot be directly transferred to top-gate TFTs. Further optimization of the annealing sequence, temperature, and duration, together with systematic evaluation of interface traps and bias-stress stability, is required to establish suitable annealing conditions for top-gate devices [50,52,53].

## 4. Conclusions

Quinary IGZTO films were grown by magnetron sputtering and then annealed in vacuum (4 × 10^−2^ Pa) at 25, 150, 250, 350 and 450 °C. Their phase constitution, surface topography, chemical bonding, optical and electrical performance were examined by XRD, SEM, AFM, XPS, UV–Vis spectrophotometry and Hall measurements, respectively. TFTs incorporating the same films were subjected to the identical annealing schedule and electrically characterized, and TCAD modelling was employed to relate the observed device behaviour to the defect statistics of the channel. The main findings can be summarized as follows.

(1) Every annealed layer remained amorphous and highly transparent, with an average visible transmittance above 83% and an optical gap between 3.22 and 3.63 eV. The gap variation is traced to two competing contributions, namely the Burstein–Moss shift controlled by the free-carrier density and the change in the degree of structural disorder. The film fired at 350 °C was both the smoothest (RMS = 294 pm, Ra = 195 pm) and the least disordered in the optical sense (*E_U_* = 0.798 eV), which we ascribe to the short-range reordering and the surface flattening that take place at this intermediate temperature.
(2) Hall measurements gave carrier concentrations between 7.44 × 10^18^ and 6.37 × 10^19^ cm^−3^ and Hall mobilities between 20.89 and 35.53 cm^2^ V^−1^ s^−1^, both depending on the annealing temperature. The temperature dependence of the carrier concentration follows that of the $V_{O}^{\times }$ density deduced from the O 1s spectra, which suggests that oxygen vacancies act as dominant shallow donors and are a major factor setting the conductivity of the IGZTO layers.
(3) Only the transistor fired at 350 °C could be switched off and on in a well-defined manner; the devices processed at 25, 150, 250 and 450 °C lacked a usable switching window because their channel contained either too many shallow acceptor-like states or an excess of newly formed defect states. At *V*_DS_ = 30 V the 350 °C device reached *μ*_Sat_ = 19.7 cm^2^ V^−1^ s^−1^, *V_TH_* = 4.72 V, *SS* = 0.56 V dec^−1^ and *I*_on_/*I*_off_ = 8.1 × 10^7^.
(4) Whether a device switches properly is governed jointly by the degree of structural disorder and by the $V_{O}^{\times }$ density. The simulations indicate that vacuum annealing promotes both the diffusion of oxygen towards the free surface and its desorption, so that the $V_{O}^{\times }$ density rises markedly in the near-surface region of the IGZTO layer. Structural order, in turn, is strongly temperature dependent. The two factors appear to reach a balance only at 350 °C—an ordered network combined with a moderate $V_{O}^{\times }$ density—and only under these conditions is normal transistor action observed. Loss of switching at 150 and 450 °C appears to be dominated by disorder, which manifests itself as steep conduction- and valence-band tails, whereas the failure at 250 °C is instead caused by an excessive $V_{O}^{\times }$ density.

However, it should be stressed that the optimal annealing condition and the conclusions proposed in the present work are based on the bottom-gate staggered IGZTO TFTs, and they cannot be extended directly to top-gate devices. The different device structure and deposition sequences of GI and the channel layer between a bottom-gate and a top-gate TFT will have a distinct impact on interface performance and oxygen exchange of GI/channel layer, which finally affects the transfer characteristics of the TFTs.

Taken together, the results suggest a practical vacuum-annealing window for quinary IGZTO channels and provide a defect-based interpretation for the performance of the bottom-gate staggered TFTs fabricated from them.

## Figures and Tables

**Table 1:** Parameters of the DOS model and material parameters of IGZTO.

Parameters	Description	Value (Bulk)	Value (Surface)	Ref.
*N_TA_*/*N_TD_*(×10^20^ cm^−3^ eV^−1^)	The defect density of acceptor-like states and donor-like states.	1.65/1.55	1.65/1.55	[28,40]
*W_TA_*/*W_TD_*(eV)	The characteristic slope energy of the conduction band tail (CBT) and valence band tail (VBT).	0.04/0.124	0.04/0.124	
$N_{V_{O}^{\times }}$(×10^16^ cm^−3^)	The defect density of neutral oxygen vacancy.	6.5	6.5	[40]
$W_{V_{O}^{\times }}$(eV)	The characteristic slope energy of the neutral oxygen vacancy.	0.046	0.046	
$N_{V_{O}^{\cdot }/V_{O}^{\cdot \cdot }}$(×10^17^ cm^−3^)	The defect density of charged oxygen vacancy.	3	7	[41]
$W_{V_{O}^{\cdot }/V_{O}^{\cdot \cdot }}$(eV)	The characteristic slope energy of the charged oxygen vacancy.	0.016	0.018	
$N_{O_{i}^{\times }}$(×10^15^ cm^−3^)	The defect density of interstitial oxygen.	1	1	[41]
$W_{O_{i}^{\times }}$(eV)	The characteristic slope energy of the interstitial oxygen.	0.01	0.01	
*D_itA_*(×10^11^ cm^−2^ eV^−1^)	The interface defect density between IGZTO and SiO_2_.	1		[28]
*W_itA_*(eV)	The characteristic slope energy of the interface defect density.	0.01		
*E_g_*(eV)	BandGap	3.42		[22,27]
*χ*(eV)	Electronic affinity	4.1		
*ε*	Dielectric constant	12		

**Table 2:** Comparison of electrical and optical properties of IGZTO films at different annealing temperatures.

*T_A_* (°C)	Electrical Properties			Optical Properties	
	*n_e_* (cm^−3^)	*μ* (cm^2^ V^−1^ s^−1^)	*ρ* (Ω·cm)	*E_g_* (eV)	*E_U_* (eV)
25	6.37 × 10^19^	20.89	4.69 × 10^−3^	3.63	1.00
150	2.97 × 10^19^	34.68	6.06 × 10^−3^	3.61	0.90
250	3.77 × 10^19^	35.53	4.65 × 10^−3^	3.22	0.88
350	7.44 × 10^18^	31.72	2.62 × 10^−2^	3.33	0.80
450	1.31 × 10^19^	33.45	1.41 × 10^−2^	3.38	1.06

**Table 3:** Electrical and optical properties of the IGZTO layers of this work against representative IGZO, IGZTO and ITZO films from the literature.

Thin Film	Method	Carrier Concentration (cm^−3^)	Hall Mobility (cm^2^/Vs)	Electrical Resistivity (Ω·cm)	Average Visible Light Transmittance	*E_g_* (eV)	Ref.
Mo:IGZO	Mc-S *	3.7 × 10^19^	25	7.75 × 10^−3^	>80%	2.95	[9]
IGZTO (150 °C)	MS	3.06 × 10^19^	35.68	5.70 × 10^−3^	>80%	3.52	[22]
IGZTO	MS	~1 × 10^16^	~25	~30	/	/	[23]
IGZTO	Sol-gel	3.48 × 10^15^	65	23.3	89%	3.66	[31]
Ga:IZO	Mc-S	1.80 × 10^20^	29	1.17 × 10^−3^	86.50%	3.40	[32]
ITZO	MS	1.27 × 10^20^	24.31	/	89.87%	3.74	[45]
IGZTO (150 °C)	MS	2.97 × 10^19^	34.68	6.06 × 10^−3^	>83%	3.61	This work
IGZTO (250 °C)	MS	3.77 × 10^19^	35.53	4.65 × 10^−3^	>83%	3.22	

* Note: MS stands for magnetron sputtering, Mc-S stands for magnetron co-sputtering, Sol–gel stands for the solution-based fabrication method.

**Table 4:** Atomic ratios of elements in IGZTO films with different annealing temperatures.

T_A_ (°C)	Atomic Percentage (at.%)				
	In (3d)	Ga (2p)	Zn (2p)	Sn (3d)	O
25	21.76	3.38	16.56	4.27	54.02
150	20.43	5.78	19.81	4	49.97
250	22.85	5.09	17.02	4.55	50.5
350	23.88	6.42	15.15	4.76	49.79
450	23.34	7.65	9.75	6.05	53.2

**Table 5:** Comparison of electrical properties of our IGZTO TFT and several representative IGZO TFT reported previously.

TFTs	Method	*μ* (cm^2^ V^−1^ s^−1^)	*V_TH_* (V)	*SS* (V/dec)	*I_on_/I_off_*	Ref.
IGZTO	MS	21.5	−0.6	0.95	5 × 10^7^	[18]
IGZTO	MS	20.17	6.19	2.60	1.1 × 10^5^	[22]
IGZTO	Sol-gel	11.5	−0.05	0.1	>10^8^	[25]
IGZTO	MS	40.4	0.5	0.49	>10^8^	[47]
IGZO	MS	13.05	0.33	0.13	1.73 × 10^8^	[50]
IGZTO	MS	26.15	2.32	0.18	/	[51]
IGZTO	MS	11.1	2.17	0.49	3.49 × 10^7^	[52]
IGZO	MS	7.64	0.62	0.11	2.29 × 10^8^	[53]
IGZTO	MS	19.7	4.72	0.56	8.1 × 10^7^	This work

Note: MS stands for magnetron sputtering, Sol–gel stands for the solution-based fabrication method.

## Data Availability

The original contributions presented in this study are included in the article. Further inquiries can be directed to the corresponding authors.
